# Clinical efficacy and regulatory mechanisms of Shi Pi Zeng Ye formula in treating functional constipation comorbid with depression: integrating clinical observation, mass spectrometry, bioinformatics, and molecular docking

**DOI:** 10.3389/fphar.2025.1645277

**Published:** 2025-08-20

**Authors:** Ling Yao, Xiaoqiang Jia, Yufei Li, Haixia Li, Zhuhui Zhang, Longfang Quan, Qiuling Liu, Jie Dai, Xuedi Lei, Huashang Li, Yonghai Li

**Affiliations:** ^1^ Department of Anorectal, Guang’anmen Hospital, China Academy of Chinese Medical Sciences, Beijing, China; ^2^ Department of Anorectal, Xi Yuan Hospital, China Academy of Chinese Medical Sciences, Beijing, China; ^3^ Department of Cardiology, Guang’anmen Hospital, China Academy of Chinese Medical Sciences, Beijing, China; ^4^ Bengbu Medical University, Anhui, China; ^5^ Department of Anorectal, The First People’s Hospital of Hefei, Hefei, China

**Keywords:** functional constipation, depression, SPZY, network pharmacology, molecular docking

## Abstract

**Ethnopharmacological relevance:**

The Shi Pi Zeng Ye Formula (SPZY), a traditional Chinese herbal compound, is empirically used for qi and yin replenishment and has been prescribed for managing functional constipation (FC) comorbid with depression. Although its clinical efficacy is recognized, the active constituents and their precise mechanisms of action in treating FC comorbid with depression have yet to be fully determined.

**Aim of the study:**

This research aims to elucidate the efficacy and mechanisms underlying the therapeutic effects of SPZY on FC comorbid with depression, employing a single-arm study design alongside mass spectrometry, network pharmacology, and molecular docking.

**Materials and methods:**

In this study, 202 patients suffering from FC were recruited and treated with SPZY over a 12-week period. The primary outcome measures included the Wexner Constipation Assessment Scale (WCS) and the Hamilton Depression Rating Scale-17 (HAMD-17). Secondary outcomes were evaluated using the Patient Assessment of Constipation Quality of Life (PAC-QOL) and the Hamilton Anxiety Rating Scale (HAMA). Assessments were conducted at baseline, 4 weeks, and 12 weeks post-treatment. The study also explored the action mechanisms of SPZY through mass spectrometry, network pharmacology, and molecular docking to ascertain the binding affinities of SPZY’s active components to critical targets.

**Results:**

The study findings indicated significant improvements in WCS (p < 0.0001), HAMD-17 (p < 0.0001), PAC-QOL (p < 0.0001), and HAMA (p < 0.001) scores from baseline to 3 months. Mass spectrometry identified Nobiletin, Tangeritin, and Magnolol as pivotal active components of SPZY. Pathological processes potentially modulated by SPZY in FC comorbid with depression include regulation of membrane potential, response to alcohol, regulation of developmental growth, and neuroactive ligand-receptor interaction pathways. Network pharmacology analysis pinpointed SLC6A3 and OPRM1 as central therapeutic targets of SPZY. Molecular docking results suggested that Sugiol, Shinpterocarpin, Medicarpin, and Formononetin have high binding affinities to SLC6A3 and OPRM1, with the SLC6A3-Medicarpin complex exhibiting the strongest binding energy (−9.6 kcal/mol).

**Conclusion:**

The SPZY formula is effective in alleviating symptoms of FC and depression. The interaction between SLC6A3 and Medicarpin is identified as a crucial mechanism in the therapeutic efficacy of SPZY for treating FC comorbid with depression.

## Graphical Abstract

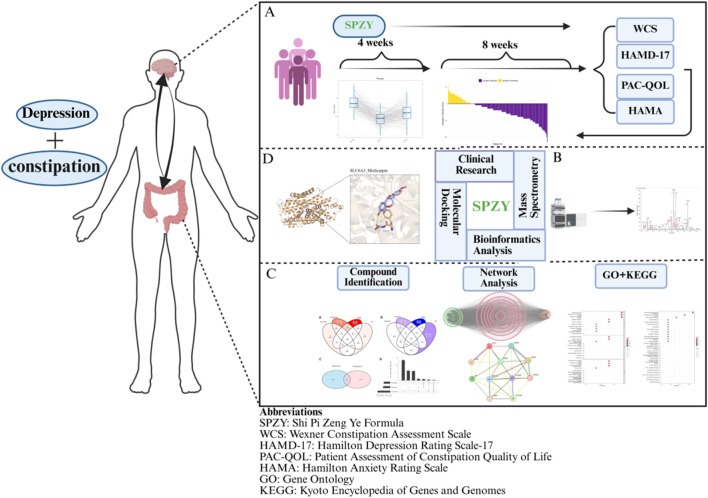

## 1 Introduction

Constipation represents a prevalent disorder that exerts a considerable burden on both individuals and healthcare systems (Werth et al., 2019). It affects approximately 18.9% of individuals aged 60 to 93 (Salari et al., 2023), and a study across multiple cities in China—Tianjin, Xiamen, Cangzhou, and Harbin—found that 17.6% of the population aged 65 and older suffer from this condition (Du et al., 2022). Notably, constipation increases the risk of hypertension and cardiovascular events in hospitalized patients over the age of 60 (Judkins et al., 2023) and is associated with a 48% increased risk of depression (Yun et al., 2024). Furthermore, research indicates that 11.31% of individuals with constipation experience depression (Wu et al., 2024). An analysis utilizing the National Health and Nutrition Examination Survey (NHANES) database revealed a significant association between constipation and major depressive disorder (OR: 2.20, p < 0.001), with odds ratios indicating that depression (OR = 11.43, p = 0.008) and major depressive disorder (OR = 1.12, p = 0.007) contribute to the likelihood of developing chronic constipation (He et al., 2024; Wang et al., 2023). The management of constipation encompasses a variety of treatments including osmotic laxatives, secretagogues, bile acid transport inhibitors, probiotics, prokinetic drugs, biofeedback therapy, and surgery (Camilleri et al., 2017; Hojo et al., 2025). However, prolonged use of laxatives may lead to adverse effects such as intestinal flora imbalance (Igudesman et al., 2023), melanosis coli (R. Zhang et al., 2023), hypokalemia (Baker and Sandle, 1996), and arrhythmias (Hoppe et al., 2019). Additionally, dependency on laxatives can impair colonic peristalsis and potentially trigger psychological disorders (Gibson et al., 2021). While biofeedback has shown limited effectiveness (Rao et al., 2018), surgical interventions report a complication rate of 24% (Knowles et al., 2017; Vriesman et al., 2020; Woodward et al., 2014). Although these treatments may alleviate the symptoms of constipation, they do not directly address underlying depressive disorders.

According to Traditional Chinese Medicine (TCM) theory, constipation and depression are not only interrelated but also stem from the same pathological process. As individuals age, there is a tendency for the spleen and kidney qi to weaken. This weakening results in a qi deficiency, which subsequently diminishes body fluid production. The consequent intestinal dryness impedes the normal propulsion of feces, thereby perpetuating a cycle of “qi failing to distribute fluids and bowel stagnation”.

TCM formulas strategically regulate the body’s yin-yang balance through multiple targets and pathways, offering significant advantages in the treatment of chronic diseases that exhibit complex complications (Meng et al., 2025). Classic prescriptions, a subset of compound prescriptions, are renowned for their stable and widely recognized therapeutic effects. For example, among the prescriptions designated for constipation, Bu Zhong Yi Qi Decoction excels in tonifying qi (Zheng et al., 2014), Zeng Ye Decoction specializes in nourishing yin (Wu et al., 2014), and Ji Chuan Decoction is adept at warming the kidney (Wang et al., 2022). However, the clinical presentation of constipation is often complex and may include multiple complications such as depression, which cannot be comprehensively addressed by a single classic prescription alone. The Shi Pi Zeng Ye Decoction (SPZY) amalgamates these three classic prescriptions to treat qi and yin deficiency constipation, representing an empirical synthesis derived from the long-term integration of traditional theories and clinical practice. SPZY includes a variety of botanical components (The botanical names of the herbs used in this study were verified using The Plant List (www.theplantlist.org) (accessed on 5 June 2025)) such as 15 g of fried Astragalus membranaceus (炙黄芪, Fabaceae); 20 g of raw Atractylodes macrocephala (生白术, Asteraceae); 10 g of Angelica sinensis (当归, Apiaceae); 15 g of Citrus reticulata (陈皮, Rutaceae); 15 g of Magnolia officinalis (厚朴, Magnoliaceae); 15 g of Cistanche deserticola (肉苁蓉, Orobanchaceae); 10 g of Scrophularia ningpoensis (玄参, Scrophulariaceae); 15 g of Rehmannia glutinosa (地黄, Orobanchaceae); 10 g of Bupleurum chinense (北柴胡, Apiaceae); 10 g of Paeonia lactiflora (白芍, Paeoniaceae); 10 g of Curcuma wenyujin (郁金, Zingiberaceae); 15 g of Prunus persica (桃仁, Rosaceae); and 6 g of Glycyrrhiza uralensis (甘草, Fabaceae). In this formula, fried Astragalus membranaceus and Atractylodes macrocephala are the main ingredients, enhancing digestive function and combating intestinal hypotonia. Prunus persica, Rehmannia glutinosa, Scrophularia ningpoensis and Cistanche deserticola increase intestinal fluid secretion. Citrus reticulata and Magnolia officinalis relieve abdominal distension, while Bupleurum chinense, Curcuma wenyujin and Paeonia lactiflora regulate emotions. Licorice coordinates the interaction of all the ingredients. These components work synergistically to boost qi and nourish yin, moisten the intestines, and relieve depression. Several herbal components within SPZY, which have established therapeutic effects on constipation and depression. For example, Atractylenolide and Atractylon found in raw Atractylodes macrocephala increase the contraction force of intestinal smooth muscle and promote peristalsis (Wu et al., 2021). Similarly, volatile oil components of Angelica sinensis stimulate the intestinal mucosa and enhance the secretion function of the intestine, softening stools (Xu et al., 2012). Additionally, Echinacoside and Cistanoside from Cistanche deserticola are known to stimulate intestinal smooth muscle, enhancing contraction strength and peristalsis frequency (Yan et al., 2017). Atractylon III demonstrates neuroprotective effects by reducing glutamate-induced neuronal apoptosis, thereby ameliorating depression (Zhou et al., 2021). Notably, the formula is free from anthraquinones, which are known to cause melanosis coli (Zhang et al., 2023).

However, robust evidence supporting the clinical efficacy of SPZY and elucidating its underlying mechanisms for treating both constipation and concurrent depressive symptoms is currently lacking. Therefore, we investigated SPZY’s efficacy and mechanisms for functional constipation (FC) with concurrent depressive symptoms using a single-arm study, mass spectrometry, and network pharmacology. Molecular docking further confirmed the stability of interactions between SPZY’s active components and their targets, supporting the hypothesized mechanisms underlying SPZY’s dual therapeutic effects.

## 2 Materials and Methods

### 2.1 Clinical trial to assess the efficacy of SPZY in treating constipation with comorbid depression symptoms

#### 2.1.1 Study subjects

This investigation employed a single-arm trial design, enrolling participants diagnosed with FC and concurrent depression symptoms, as indicated by a Hamilton Depression Rating Scale (HAMD)-17 score of eight or higher (Leucht et al., 2013). Recruitment occurred at the Hefei First People’s Hospital between 1 January 2024 and 17 February 2025. All participants met predefined inclusion and exclusion criteria. The Ethics Committee of Hefei First People’s Hospital granted approval for this trial (Approval No.: 2024-181-01).

**FIGURE 1:**
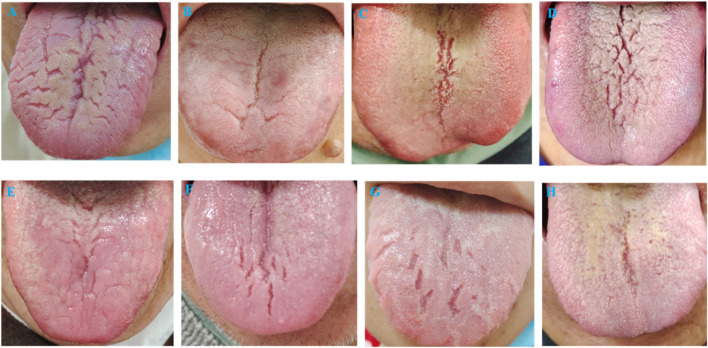
Typical tongue manifestations of qi and yin deficiency. **(A–H)** shows red tongue body with cracks and scanty coating, indicating qi and yin deficiency.

FC was defined according to the Rome IV diagnostic criteria (Zhang et al., 2021). These criteria necessitate the exclusion of constipation caused by intestinal and systemic organic pathologies, medications, and other factors. The diagnostic criteria are met if the symptoms have persisted for at least 6 months prior to diagnosis, with the last 3 months conforming to the following specifications: (1) experiencing at least two of the specified symptoms; (2) infrequent loose stools in the absence of laxatives; (3) non-conformity to the diagnostic criteria for IBS-C (IBS-C (Constipation-predominant Irritable Bowel Syndrome): A specific subtype of irritable bowel syndrome characterized primarily by constipation (difficulty defecating, reduced bowel frequency, hard or lumpy stools) accompanied by recurrent abdominal pain. The abdominal pain typically alleviates following defecation.). The specific symptoms include: (1) difficulty in defecation occurring at least 25% of the time; (2) lumpy or hard stools at least 25% of the time; (3) sensations of incomplete evacuation at least 25% of the time; (4) feelings of anal or rectal blockage at least 25% of the time; (5) the necessity for manual assistance during defecation at least 25% of the time, such as using fingers for fecal evacuation or pelvic floor support; (6) fewer than three spontaneous bowel movements per week. The syndromes of Qi and Yin deficiency were characterized as follows (Zhang, 2017): Qi deficiency syndrome: Primary symptoms include weakness during defecation and dull abdominal pain alleviated by massage. Secondary symptoms encompass fatigue, diminished verbal interaction, and appetite loss. Diagnostic physical signs are a pale red tongue, possibly swollen or marked by teeth, with a thin white coating and a weak pulse. Yin deficiency constipation: Primary symptoms are hard stools resembling sheep droppings and persistent dry mouth paired with thirst. Secondary symptoms cover heat sensations in the palms and soles, weight loss, irritability, and insomnia. The tongue and pulse manifestation involves a red tongue with cracks, thin coating, and a fine pulse. A diagnosis mandates at least one primary symptom of both Qi and Yin deficiency, one secondary symptom, and one indicative sign from tongue and pulse examinations (refer to Figure 1 for typical tongue coatings).

The criteria required for inclusion are as follows: (1) Fulfilling the diagnostic criteria for FC as outlined in Rome IV, meeting the symptom criteria for qi and yin deficiency in TCM, and possessing a HAMD score greater than 7; (2) Aged between 18 and 75 years; (3) Demonstrating normal comprehension and possessing the ability to read and write; (4) Providing signed informed consent. Individuals will be excluded from participating in the study based on the following criteria: (1) Presence of severe functional disorders in critical organs or systems such as the heart, brain, liver, kidneys, blood, or endocrine system; (2) Current use of medications known to induce constipation; (3) Status as a pregnant or lactating woman; (4) Inability to attend regular follow-up visits; (5) Concurrent participation in other clinical trials.

#### 2.1.2 Intervention

The SPZY granules were prepared and provided by the pharmacy at Hefei First People’s Hospital and sourced from Hebei Baocao Kangshen Pharmaceutical Co., Ltd. Participants are to consume the granules dissolved in boiling water twice daily. Each treatment course lasts 4 weeks, with a total of three courses. During this period, only SPZY is to be taken. The use of a glycerin enema is permitted as an auxiliary measure when spontaneous defecation does not occur.

#### 2.1.3 Sample size calculation

The study adopts a single-arm superiority design, utilizing a hypothesis-testing framework for binary endpoints to evaluate efficacy. Drawing on prior research (Elkaragy et al., 2024), the efficacy of the standard treatment is assumed to be 70% (P0), while the target efficacy (P_T_) for the intervention is set at a conservative 84%, indicating a superiority margin (Δ) of 14%. The specified parameters for the statistical analysis include: A significance level (α) of 0.05 (two-sided test); a power (1 - β) of 99.5%; an anticipated dropout rate of 15%. The sample size calculation formula is (Chow et al., 2003).

$$n=\frac{{\left[\sqrt{Z_{1-a/2}P_{0}\left(1-P_{0}\right)}Z_{1-\beta }\sqrt{P_{T}\left(1-P_{T}\right)}\right]}^{2}}{{\left(P_{T}-P_{0}\right)}^{2}}\times \left(1+dropout\;rate\right)$$

n = sample size; Z_1-α_ and Z_1-β_ = standard normal distribution quantiles; *P*_0_ = assumed standard efficacy rate (70%); *P*_*T*_ = target intervention efficacy rate (84%); α = Type Ⅰ error rate (0.05)→Z_1-α_ = 1.96; β = Type II error rate (0.005)→Z_1-β_ = 2.576.

Using these parameters, the initial calculated sample size is approximately 173 participants. Adjusting for the expected dropout rate, the total sample size required is approximately 200 participants.

#### 2.1.4 Outcome measures

##### 2.1.4.1 Primary outcome measures

Wexner Constipation Assessment Scale (WCS): Developed by Feran Agachan and colleagues (Agachan et al., 1996), the WCS is a validated scoring system designed to quantify the severity of constipation. It comprises eight components: frequency of defecation, pain during defecation, sensation of incomplete evacuation, abdominal pain, average time per defecation, use of assistance methods during defecation, the number of unsuccessful defecation attempts within 24 h, and duration of the condition. For further details, refer to Supplementary Appendix S1. The scale will be administered at baseline, 4 weeks and 12 weeks post-intervention.

HAMD-17: Originally developed by Hamilton and later refined by Ma and colleagues (Ma et al., 2021), the HAMD-17 is a widely recognized clinician-administered scale for assessing depressive symptoms. Following adequate training, researchers will conduct these assessments within 1 week of each scheduled observation point. For details, see Supplementary Appendix S1. The scale has a maximum score of 52, with depression severity categorized as mild (>7, ≤17), moderate (>17, ≤24), and severe (>24), based on the score achieved. This scale focuses on symptoms experienced in the 2 weeks leading up to each assessment. The HAMD-17 will be utilized at baseline, 4 and 12 weeks post-intervention.

##### 2.1.4.2 Secondary outcome measures

Patient Assessment of Constipation Quality of Life (PAC-QOL): Endorsed by the Mapi Research Trust (Marquis et al., 2005), the PAC-QOL is a validated self-report instrument that evaluates the impact of constipation on a patient’s daily life and overall wellbeing. It includes 28 items that assess physical and psychological discomfort, worry, and satisfaction with treatment. For additional information, please consult Supplementary Appendix S3. This assessment is scheduled to be conducted at baseline, 4 and 12 weeks following the intervention.

The Hamilton Anxiety Rating Scale (HAMA) (Maier et al., 1988) is widely recognized as a fundamental clinician-administered instrument for evaluating anxiety in clinical settings. It is particularly adept at quantifying the severity of anxiety symptoms. The HAMA scale comprises 14 items categorized into somatic and psychic domains. For further information, refer to Supplementary Appendix S4. The evaluation encompasses symptoms exhibited during the fortnight preceding the assessment. Measurements were conducted at baseline, 4 and 12 weeks post-intervention.

##### 2.1.4.3 Quality control

For data collection, self-report questionnaires (WCS, PAC-QOL) were administered using “Questionnaire Star”, a widely used online survey and data collection platform in China. Minimum response times were established (e.g., WCS ≥3 min, PAC-QOL ≥5 min) to ensure the thoroughness of responses. Participants were required to complete the questionnaires within a 2-day period.

For other report questionnaires (Hamilton Anxiety Scale, 17-item Hamilton Depression Scale), assessor Ling Yao received comprehensive training at the Beijing Sixth Hospital (The World Health Organization (WHO) Collaborating Center for Mental Health Research and Training in Beijing) for 3 months. This included 16 h of structured workshops using standardized case summaries, followed by a competency assessment requiring an accuracy rate of ≥90% in 20 validation cases. Reliability tests were conducted: the intraclass correlation coefficient of the 17-item Hamilton Depression Scale reached 0.85, and that of the Hamilton Anxiety Scale reached 0.82 (both exceeding the clinical reliability standard of 0.8 (Koo and Li, 2016)). Throughout the trial, 10% of the assessment results were randomly selected for blind re-evaluation by a senior psychiatrist, and any differences were resolved through consensus discussion.

#### 2.1.5 Statistical methods

Statistical analyses were performed using SPSS version 25.0 (IBM Corporation, Chicago, IL, USA). Data were presented as mean ± SD. Repeated Measures Analysis of Variance (ANOVA) was employed to compare the differences in WCS scores, PAC-QOL scores, HAMD-17 scores, and HAMA scores before and after treatment. A p-value of less than 0.05 was deemed to indicate statistical significance. Additionally, the R programming language (version 4.5.0) was utilized for graphical presentations.

## 3 New active substances in SPZY analyzed by mass spectrometry SPZY

### 3.1 Instruments

The array of experimental instruments comprised the following: a TQ-Exactive HFX mass spectrometer and a UHPLC Vanquish ultra-high performance liquid chromatography system (both from Thermo Fisher Scientific, Bremen, Germany) used primarily for sample analysis. Sample processing was facilitated by an Eppendorf Centrifuge 5430 R (Eppendorf, Hamburg, Germany), an FD-1C-80 freeze dryer (Shanghai Bilon Instruments Co., Ltd., Shanghai, China), and an SB-4200D ultrasonic cleaner (Ningbo Scientz Biotechnology Co., Ltd., Ningbo, China). Additionally, a ME104 electronic balance (Mettler Toledo, Shanghai, China) and a QT-1 vortex mixer (Xiexu Medical, Shanghai, China) were employed.

### 3.2 Mass spectrometry process

#### 3.2.1 Analytical method

The chemical constituents of SPZY were rigorously characterized through ultra-high performance liquid chromatography coupled with high-resolution mass spectrometry (UHPLC-Q-Exactive HFX, Thermo Fisher Scientific). Chromatographic separation was executed on an HSS T3 column (2.1 × 100 mm, 1.8 μm, 100 Å pore size) employing a gradient elution of 0.1% (v/v) aqueous formic acid and acetonitrile at a flow rate of 0.3 mL/min. Mass spectrometric detection was performed in both positive and negative electrospray ionization (ESI) modes, utilizing a full-scan/data-dependent MS^2^ (dd-MS^2^) acquisition strategy. The critical parameters included a primary mass resolution of 60,000, a secondary resolution of 15,000, and stepped collision energies of 20, 40, and 60 eV.

#### 3.2.2 Sample preparation

SPZY granules were finely ground at room temperature. Subsequently, 100 mg of the finely homogenized powder was precisely measured and placed into a 1.5 mL microcentrifuge tube. The sample was then extracted using 1 mL of 70% methanol aqueous solution, subjected to vortex mixing for 30 s, followed by ultrasonic-assisted extraction (600 W, 40 kHz, 4°C, 30 min). Post-extraction, the mixture was centrifuged (16,000×g, 4°C, 10 min), and the supernatant was filtered through a 96-well protein-binding plate under positive-pressure nitrogen before being transferred to a 2 mL conical Eppendorf tube (This step efficiently adsorbed residual proteins and phospholipids, enhancing MS detection sensitivity by reducing matrix interference while ensuring high-throughput reproducibility.). The filtrate was subsequently lyophilized, reconstituted in 400 μL of 40% (v/v) methanol-water, thoroughly mixed by vortexing (30 s), and centrifuged (16,000×g, 4°C, 10 min) in preparation for UHPLC-MS analysis.

#### 3.2.3 Data processing

The raw data files (.raw) were converted into mzXML format utilizing ProteoWizard. This was followed by peak alignment, retention time correction, and feature extraction using XCMS. Compound identification was successfully achieved by querying a custom-built TCM database, followed by quantitative analysis and taxonomic classification of the annotated components. For detailed information on the mass spectrometry process, please refer to the Supplementary Appendix S5.

## 4 Network pharmacological analysis of the potential mechanism of SPZY in treating FC combined with depressive symptoms

### 4.1 Acquisition of chemical components and targets of SPZY and constipation and depression targets

Using the TCMSP Database - TCM Systems Pharmacology Database and Analysis Platform (http://tcmspw.com/tcmsp.php), we entered the names of the following herbs sequentially under the “Herb name” search category:Astragalus membranaceus; Atractylodes macrocephala; Angelica sinensis; Citrus reticulata; Magnolia officinalis; Cistanche deserticola; Scrophularia ningpoensis; Rehmannia glutinosa; Bupleurum chinense; Paeonia lactiflora; Curcuma wenyujin; Prunus persica and Glycyrrhiza uralensis. The results were filtered for oral bioavailability (OB) ≥ 30% and drug-likeness (DL) ≥ 0.18 to identify relevant chemical components of SPZY (Huang et al., 2017; Tao et al., 2020). Subsequently, under the sections “Related Targets” and “Targets Information”, we queried the target proteins of these components, standardizing the gene names of these proteins by importing them into the UniProt database (https://www.uniprot.org/).

The terms “FC” and “depression” were independently searched in the following databases: Gene Cards (https://www.genecards.org/), OMIM (https://www.omim.org/), PharmGKB (https://www.pharmgkb.org/), TTD (http://db.idrblab.net/ttd/), and DisGeNET (https://www.disgenet.org/). The collected target genes were then standardized by importing them into the UniProt database. We eliminated duplicate target genes from the effective components of SPZY and those associated with constipation and depression. A Venn diagram illustrating the overlapping target genes among constipation, depression, and SPZY was created using the “venn” package in R.

### 4.2 Construction of the drug component-dual disease target network and the drug component-disease target protein-protein interaction (PPI) network

We selected the intersecting targets from the Venn diagram that linked SPZY with FC and depression. Utilizing the Mol IDs of these intersecting targets and the effective component targets identified in the “Results” section, we generated network and tape files. These were uploaded to Cytoscape software version 3.10.3 (https://cytoscape.org/) to establish the target network for SPZY’s effective components in relation to constipation and depression.

The component-disease intersecting targets were then imported into the “Multiple Proteins” module of the STRING database (https://stringdb.org/) with the organism filter set to “*Homo sapiens*”. PPI analysis was conducted with a minimum interaction score threshold of 0.4 (Debnath et al., 2025), applying the settings interface to systematically exclude disconnected nodes, thus constructing the PPI network. The resulting TSV-formatted interaction data were imported into Cytoscape software version 3.7.2 (National Institute of General Medical Sciences). Network centrality analysis was performed using the CytoNCA 2.1.6 plugin. Core targets were identified by applying a tripartite screening criteria: (1) degree centrality, (2) closeness centrality, and (3) betweenness centrality. Nodes exceeding the median values in all three criteria were retained as biologically significant core targets.

### 4.3 Gene ontology (GO) enrichment analysis and KEGG pathway analysis

In this study, the shared targets of SPZY, FC, and depression were examined through GO and KEGG enrichment analyses, employing the “clusterProfiler” package in R. The enrichGO function (parameters: p value Cutoff = 1, q value Cutoff = 1, ont = “all”) annotated the genes across the domains of biological processes (BP), cellular components (CC), and molecular functions (MF). Only significant entries (p < 0.05, q < 0.05) were retained, and the top ten were displayed using facet bar charts and bubble charts. For KEGG analysis, the enrichKEGG function (organism = “has”) identified significant pathways (p < 0.05, q < 0.05). Gene symbols were subsequently converted, and the principal pathways were depicted through bar charts, bubble charts, and pathview pathway heatmaps. The significance of enrichment was assessed using the hypergeometric test, with entries retaining a p. adjust <0.05 and an enrichment factor >1 after applying the Benjamini–Hochberg correction. This analysis systematically elucidated the biological functions and pathway associations of the common targets.

## 5 Molecular docking

Initially, the target protein receptor’s PDB format file was obtained from the RCSB Protein Data Bank (RCSB PDB) (https://www.rcsb.org/), and the 2D structure of SPZY’s active components was sourced from PubChem (https://pubchem.ncbi.nlm.nih.gov/). Water molecules and ligands were subsequently removed from the protein receptor using PyMOL. The PDBQT file was then created by applying hydrogenation treatment and assigning charges using the Gasteiger method in AutoDock Tools (version 1.1.2; Scripps Research Institute, USA). The ligand structure underwent minimization utilizing the MMFF94 force field in Chem3D. Molecular docking was conducted with AutoDock Vina, where the binding affinity was gauged based on the calculated free energy (ΔG). The top five compounds were selected based on the most negative ΔG values. Consistent with prior research (Bissantz et al., 2010; Kollman et al., 2000), the more negative the binding free energy, the stronger the binding capacity. Weak binding is indicated by a binding free energy of approximately −5 kcal/mol, moderate binding by −8 to −10 kcal/mol, and strong binding by less than −12 kcal/mol.

## 6 Results

### 6.1 Clinical study

#### 6.1.1 Baseline characteristics

The study commenced with 224 participants. After a 4-week intervention period, exclusions were made for participants who submitted invalid questionnaires (n = 3), those lost to follow-up (n = 5), those demonstrating poor medication compliance (n = 4), and individuals concurrently using other laxatives (n = 4). This resulted in 208 participants being eligible for further analysis. Following a 12-week intervention period, additional exclusions were made for participants with invalid questionnaires (n = 2), those lost to follow-up (n = 1), those with poor medication compliance (n = 1), and those using other laxatives (n = 2), leaving 202 participants with complete data available for final analysis. Baseline characteristics of participants are detailed in Table 1.

**TABLE 1:** Demographic and clinical characteristics of enrolled patients.

Variables	SPZY (N = 202)
Demographic characteristics	
Age (mean ± SD)	56 ± 12
Gender (Female) (n/%)	143 (70.79)
Clinical characteristics	
Constipation duration (years, mean ± SD)	5.2 ± 3.1
Family history of constipation (n/%)	77 (38.12)
Causes of constipation (n/%)	
Unhealthy lifestyle habits	71 (35.15)
Pregnancy	22 (10.89)
Intestinal surgery	14 (6.93)
Fecal withholding	28 (13.86)
Medication use	40 (19.31)
Comorbidities (n/%)	
Depression*	
Mild (7-17 points)	88 (43.56)
Moderate (18-24 points)	82 (40.59)
Severe (>24 points)	32 (15.84)
Anxiety	54 (26.73)
Insomnia	90 (44.60)
Parkinson’s disease	5 (2.48)
Hypothyroidism	7 (3.47)
Coronary heart disease	38 (18.80)
Diabetes mellitus	21 (10.40)

Note: * The severity of depression is categorized based on the Hamilton Depression Rating Scale (HAMD) criteria. unhealthy life habits include inadequate dietary fiber intake; chronic dehydration; sedentary behavior; habitual suppression of defecation urges; excessive consumption of processed foods, dairy products, and red meat; overreliance on laxatives; irregular meal patterns.

#### 6.1.2 Outcome

**FIGURE 2:**
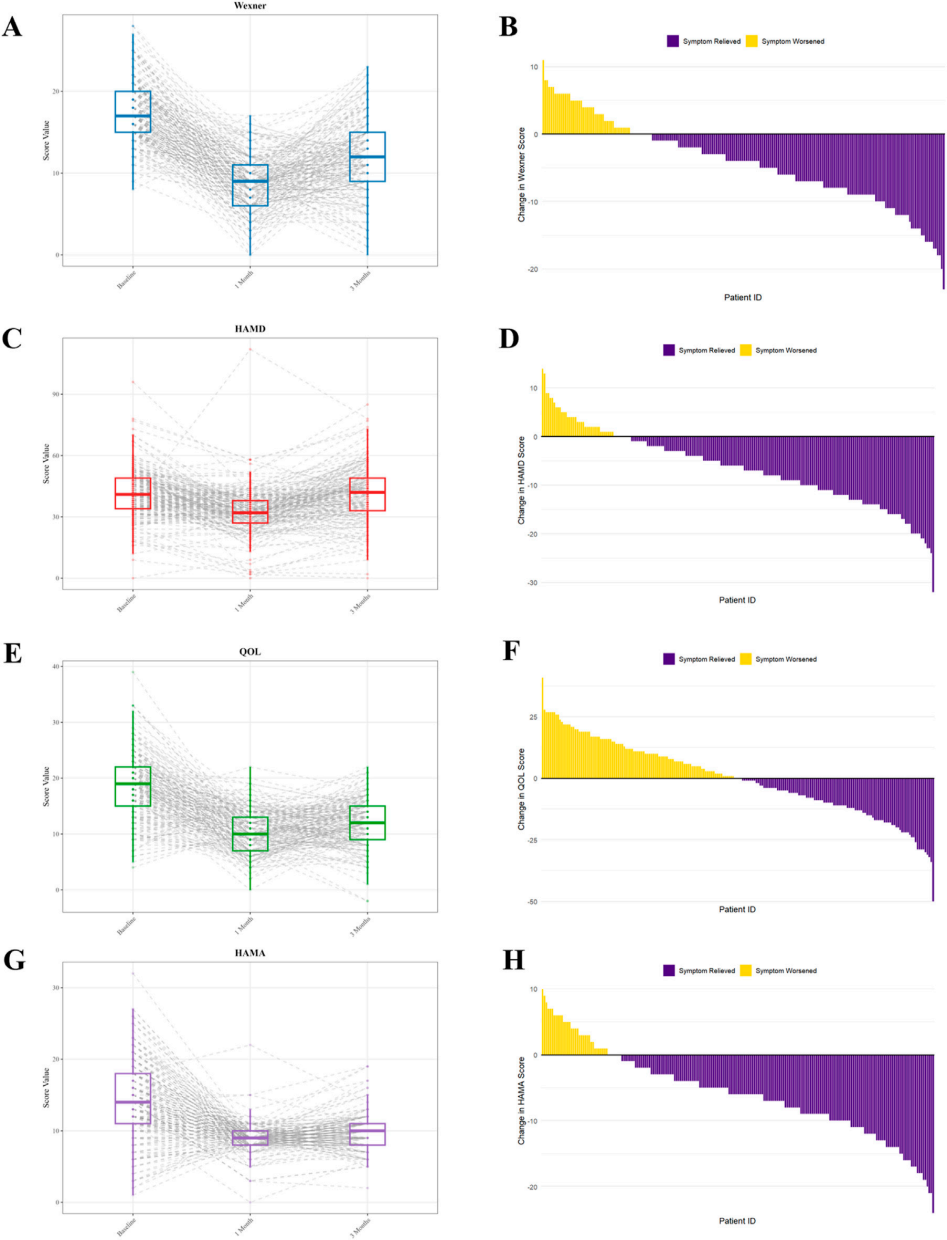
WCS score, HAMD-17, PAC-QOL, and HAMA scores before and after SPZY intervention. **(A, C, E, G)** represent the score changes of the four scales (WCS, HAMD-17, PAC-QOL, and HAMA) at baseline, 4 weeks and 12 weeks after intervention; **(B, D, F, H)** represent the differences in the scores of the four scales at 3 months after intervention compared with those before intervention (negative values indicate alleviation of constipation/depression/anxiety symptoms or improvement in quality of life). The horizontal line inside the box represents the median. The lower boundary of the box indicates the lower quartile (Q1, 25th percentile), and the upper boundary of the box indicates the upper quartile (Q3, 75th percentile). WCS, Wexner Constipation Scale; HAMD-17, 17-item Hamilton Depression Rating Scale; PAC-QOL, Patient Assessment of Constipation Quality of Life; HAMA, Hamilton Anxiety Rating Scale. SPZY, Shi Pi Zeng Ye formula.

Figure 2 illustrates the pre- and post-intervention values for the WCS score, HAMD-17, PAC-QOL, and HAMA, as well as the differences between the scores of these four scales at 3 months post-intervention compared to baseline.

The initial mean WCS score was 12.58 ± 7.12, which decreased to 8.31 ± 4.84 1 month post-intervention and further to 8.73 ± 5.16 after 3 months. These scores adhered to a normal distribution (p > 0.05) and exhibited homogeneity of variances (p = 0.243). ANOVA revealed a significant effect of time on the WCS score (F (2, 402) = 148.56, p < 0.0001). Post hoc comparisons showed significant improvements from baseline to 1 month (p < 0.0001) and from baseline to 3 months (p < 0.0001), with stability between one and 3 months (p = 0.243). These findings indicate that the intervention significantly ameliorated symptoms at 1 month, with sustained effects at 3 months.

The baseline HAMD score averaged 23.15 ± 8.67, which reduced to 15.42 ± 6.93 after 1 month of intervention, and further to 12.78 ± 5.84 after 3 months. These scores were normally distributed (p > 0.05) and exhibited variance homogeneity (p = 0.187). The ANOVA results demonstrated a significant time effect on HAMD scores (F (2, 398) = 89.24, p < 0.0001). Post hoc comparisons revealed significant reductions from baseline to 1 month (p < 0.0001), baseline to 3 months (p < 0.0001), and one to 3 months (p = 0.001), suggesting that the intervention markedly improved symptoms after 1 month, with further improvements observed at 3 months.

The initial mean PAC-QOL score was 45.89 ± 16.32. Following 1 month of intervention, this score decreased to 36.78 ± 15.21, and after 3 months, it adjusted to 42.56 ± 17.43. Both scores complied with the criteria for a normal distribution (p > 0.05) and demonstrated homogeneity of variance (p = 0.243). A variance analysis revealed a significant influence of the time factor on the Wexner score (F (2, 402) = 148.56, p < 0.0001), with the following specific comparisons: baseline versus 1 month (p < 0.0001), baseline versus 3 months (p < 0.0001), and 1 month versus 3 months (p = 0.243). These results suggest that the interventions markedly improved symptoms at 1 month, with the therapeutic effects stabilizing by 3 months.

The average baseline HAMA score was 16.24 ± 6.85, which decreased to 8.94 ± 4.37 after 1 month of intervention, and adjusted to 11.03 ± 5.62 after 3 months. The data at each time point conformed to a normal distribution (p > 0.05) and exhibited equality of variances (p = 0.318). An ANOVA indicated a significant main effect of time on the HAMA scores (F (2, 402) = 89.74, p < 0.001). Post hoc comparisons revealed significant reductions from the baseline to 1 month (p < 0.001) and from the baseline to 3 months (p < 0.001), while the comparison between 1 month and 3 months was not statistically significant (p = 0.107). These findings suggest that the intervention led to significant symptom improvement after 1 month, with the effects remaining stable thereafter.

### 6.2 Mass spectrometry results

**FIGURE 3:**
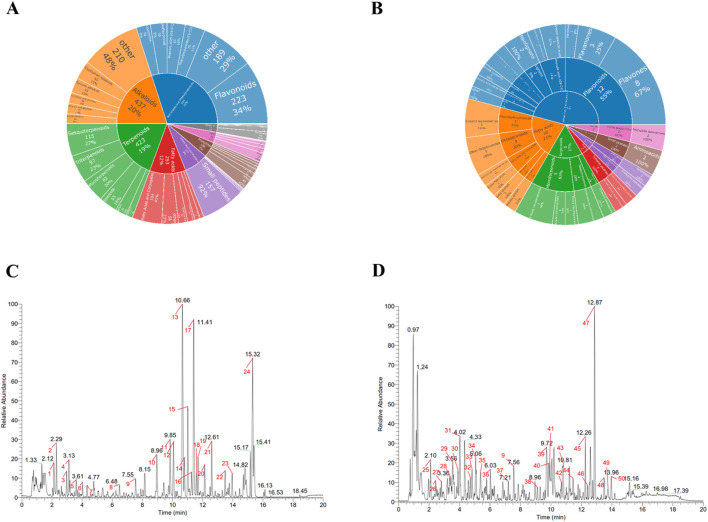
Chemical Classification and Base Peak Chromatogram Analysis of SPZY Compounds **(A)** Proportional distribution of all identified compounds (n = 2,362) across chemical classes **(B)** Proportional distribution of annotated chromatographic peaks (n = 50) across chemical classes **(C)** Base Peak Chromatogram Chart of SPZY in Positive Ion Mode - Marked Peaks **(D)** Base Peak Chromatogram Chart of SPZY in Negative Ion Mode - Marked Peaks The compounds identified in this experiment (including those identified by both positive and negative ions) were annotated with compound categories using the NPClassifier classification method. NPClassifier is a structure classification tool based on deep neural networks, specifically designed for natural products (such as plants and microorganisms). It automatically classifies natural products (NPs) based on their structural descriptors - counted Morgan fingerprints (CMFs) - to help understand the molecular structure, chemical properties, biological activities, and biosynthetic pathways of natural products. The classification system of NPClassifier divides the structures of natural products into three levels: seven pathways, 70 superclasses, and 672 classes. Pathway is used to denote different biosynthetic pathways; Superclass represents subcategories of Pathway, signifying general categories of metabolites, general chemical/molecular shapes, or biosynthetic information. Class is further subdivided from Superclass, representing specific compound families, characteristic functional groups, or skeletal diversity within a Superclass. **(A)** Classification of compounds identified by SPZY, including top five categories of pathway and superclass. **(B)** Classification of compounds identified by SPZY peak labeling, including top five categories of pathway and superclass as well as top three categories of class. The 50 annotated peaks represent chromatographically prominent constituents selected via intensity thresholds, peak purity evaluation, and identification confidence (mass error <25 ppm; MS/MS score >0.7). SPZY, Shi Pi Zeng Ye formula.

We analyzed the distribution of identified compounds across chemical classes. Figure 3A illustrates the distribution of all 2,362 identified compounds, with flavonoids (34%) being the most abundant class, followed by alkaloids (20%) and terpenoids (19%). Figure 3B details the classification of the 50 compounds identified from chromatographic peaks, where flavonoids (25.30%), fatty acids (21.0%), and terpenoids (17%) represented the primary constituents.

Base Peak Chromatograms (BPC) of the positive and negative ions of SPZY are depicted in Figures 3C,D, respectively. A total of 50 chromatographic peaks were annotated, with flavonoids, alkaloids, and phenylpropanoids constituting a significant proportion of the identified compounds, including Nobiletin (Flavonoids, m/z 403.1387, 10.66), Tangeritin (Flavonoids, m/z 373.1281, 11.41), and Magnolol (Lignans, m/z 265.1231, 12.87). Pearson correlation analysis confirmed high correlation coefficients (>0.9) among samples, indicating excellent repeatability and stability. The methods and key results of mass spectrometry are documented in Supplementary Appendix S5. For comprehensive details regarding all detected compounds, please refer to Supplementary Table S1.

### 6.3 Network pharmacology results

#### 6.3.1 Acquisition of drug and disease targets

**FIGURE 4:**
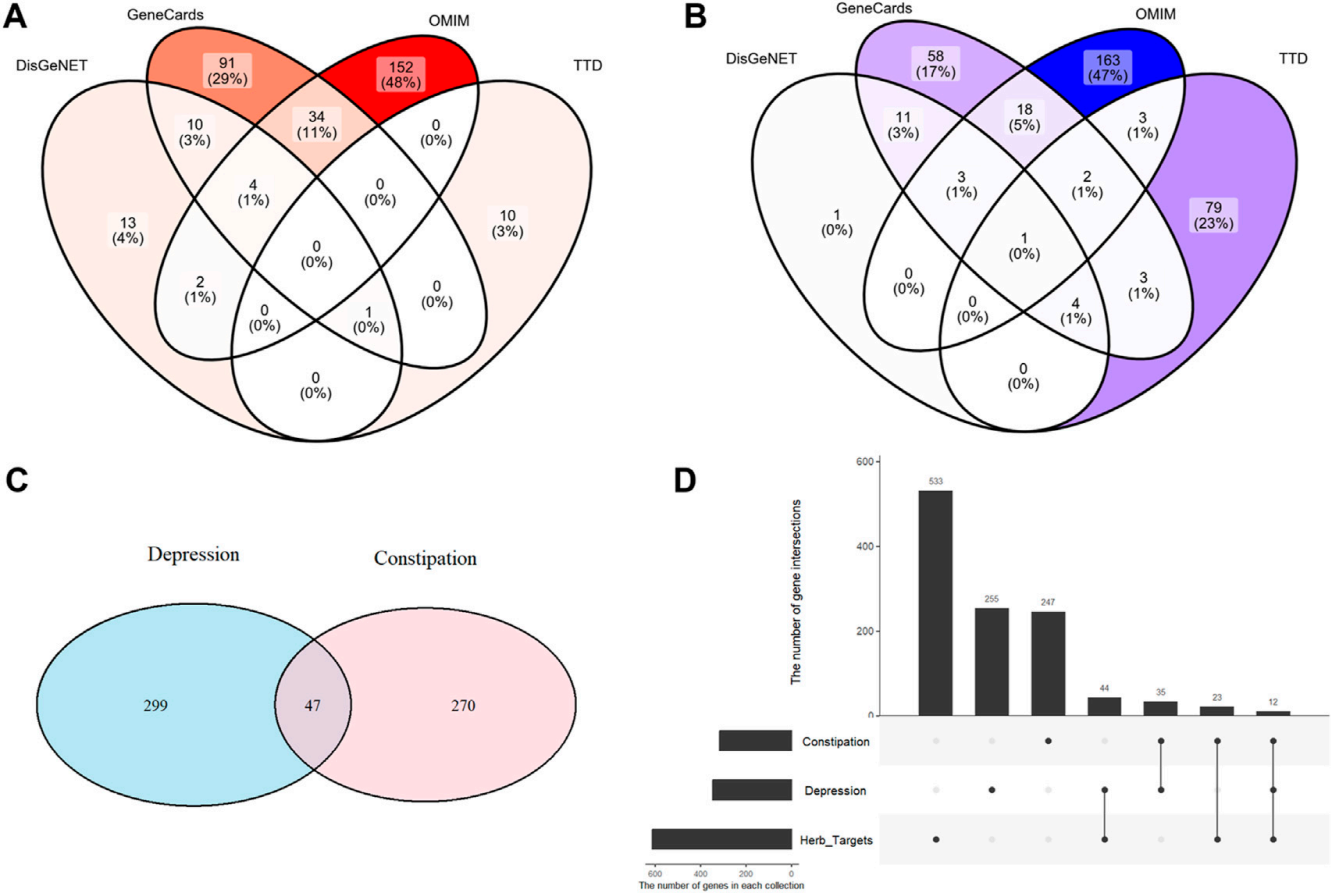
Intersection of target genes for FC and depression diseases and target genes of SPZY drug components. Union of FC target genes in Gene Cards, OMIM, TTD, and DisGeNET **(A)** union of depression target genes **(B)** intersection of depression and FC disease target genes **(C)** intersection of FC and depression target genes with SPZY drug target genes **(D)**.

Initially, a comprehensive analysis of drug targets associated with SPZY yielded 9,952 targets from the TCMSP database. Concurrently, disease targets for FC and depression were compiled from several databases, including DisGeNET, GeneCards, OMIM, and TTD. After the elimination of duplicates, 270 potential targets for FC (Figure 4A) and 299 for depression were identified, culminating in the discovery of 47 common targets between the two conditions (Figure 4B). Subsequently, a Venn diagram illustrating the overlap between these disease targets was created using R language (Figure 4C). Additionally, an analysis of the intersection of targets for FC, depression, and SPZY revealed 12 shared targets across all three categories (Figure 4D).

#### 6.3.2 Construction of drug-active component-target-disease topological network, PPI network and screening of core targets

**FIGURE 5:**
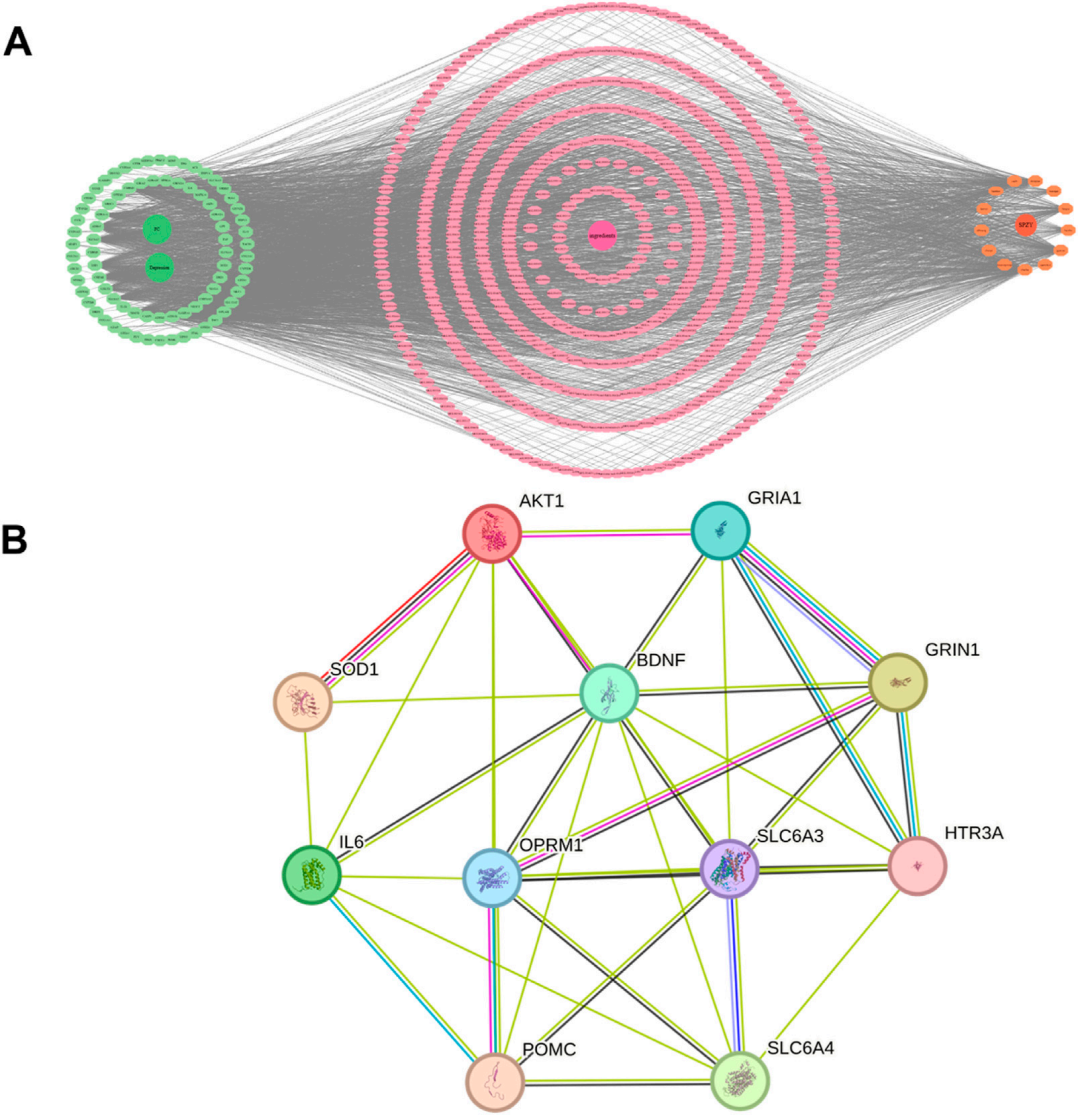
Topological network diagram **(A)** and PPI network **(B)** of the relationship between the active ingredients, targets of SPZY and diseases (FC and depression). Note: **(A)** The green circles on the left represent the common targets of the drug and the diseases. The deep green circles in the center represent functional constipation (FC) and depression respectively. The pink indicates each active compounds of SPZY that have interaction relationships with two disease targets. The deep pink circle in the center represent the active compounds of SPZY that have interaction relationships with the two disease targets. The orange circles represent the 13 medicinal plants of SPZY formula. The deep orange circle represents SPZY. FC, functional constipation; SPZY, Shi Pi Zeng Ye formula. AKT1, RAC-alpha serine/threonine-protein kinase; SOD1, Superoxide dismutase [Cu-Zn]; SLC6A, Sodium-dependent dopamine transporter; POMC, Pro-opiomelanocortin; IL6, Interleukin-6; OPRM1, Mu-type opioid receptor; BDNF, Neurotrophic factor BDNF precursor form; GRIA1, Glutamate receptor 1; SLC6A4, Sodium-dependent serotonin transporter; HTR3A, 5-hydroxytryptamine receptor 3A; GRIN1, G protein-regulated inducer of neurite outgrowth 1.

The topological network mapping drug-active components, targets, and diseases was constructed using Cytoscape v3.9.0, as depicted in Figure 5A. In this network, the right side features orange circles representing the active ingredients of SPZY, including Astragalus membranaceus; Atractylodes macrocephala; Angelica sinensis; Citrus reticulata; Magnolia officinalis; Cistanche deserticola; Scrophularia ningpoensis; Rehmannia glutinosa; Bupleurum chinense; Paeonia lactiflora; Curcuma wenyujin; Prunus persica; and Glycyrrhiza uralensis. The central deep orange circle symbolizes SPZY itself. The red circles in the middle represent the active components of SPZY, while the green circles on the left denote the common targets of the drugs and diseases, with deeper green circles in the center representing FC and depression.

Utilizing the STRING database, 11 core targets were identified: AKT1, SOD1, SLC6A3, POMC, IL6, OPRM1, BDNF, GRIA1, SLC6A4, HTR3A, and GRIN1(Network Metrics for 11 Genes in Supplementary Table S2). These were used to construct the core target network (Figure 5B). Further refinement was conducted to identify core targets whose index values for Betweenness, Closeness, Degree exceeded the median values for these metrics. This additional analysis determined that SLC6A3 and OPRM1 fulfilled these criteria (Network Metrics for Genes SLC6A3 and OPRM1in Supplementary Table S3).

#### 6.3.3 GO and KEGG enrichment analysis

**FIGURE 6:**
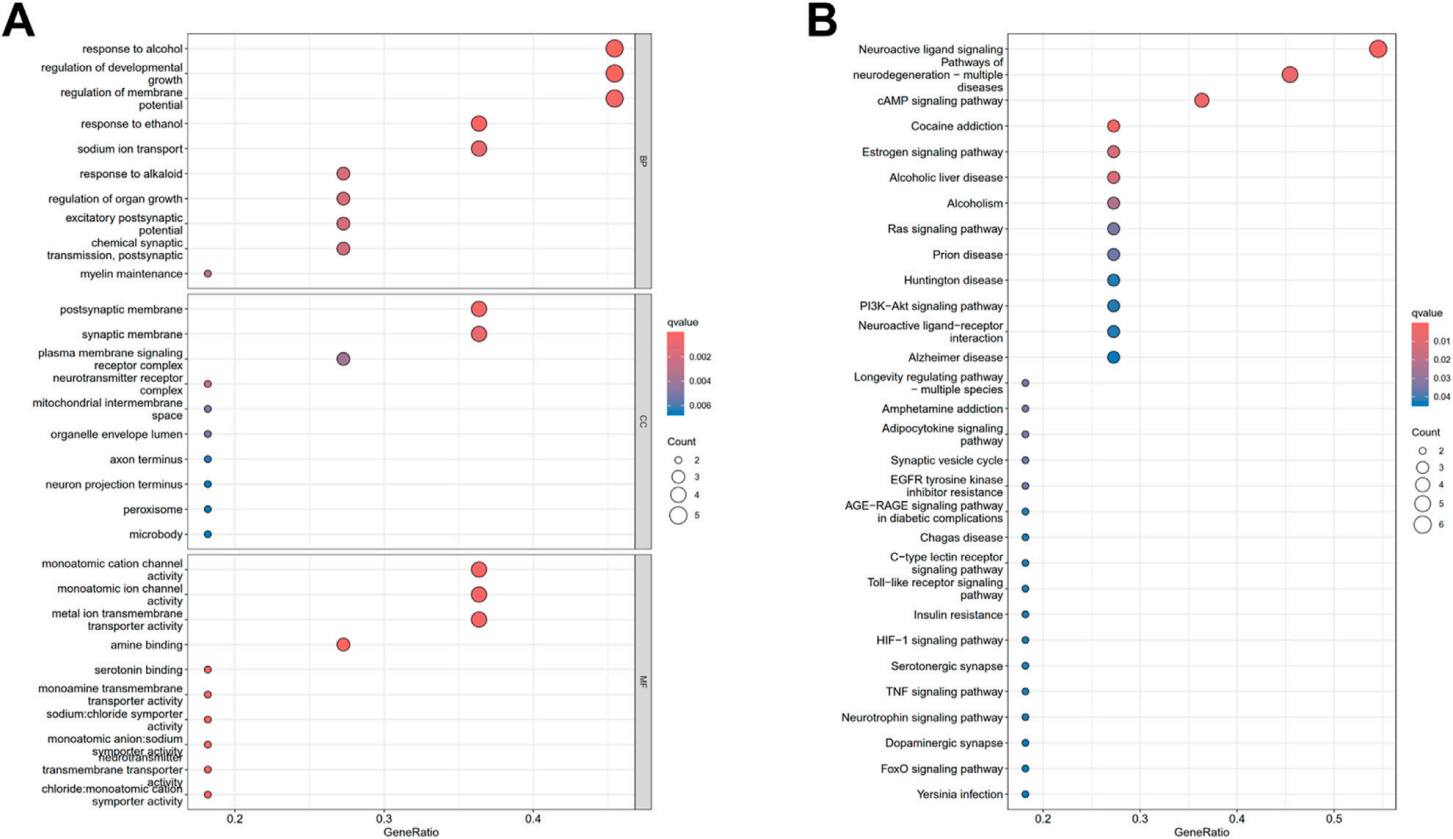
Gene Ontology enrichment analysis **(A)** and KEGG pathway analysis **(B)** of the potential therapeutic targets of SPZY in the treatment of functional constipation (FC) comorbid with depression.

The results of the GO enrichment analysis are depicted in Figure 6A. In the category of BP, a total of 721 terms were identified as significantly enriched. These primarily include terms such as “response to alcohol”, “regulation of developmental growth”, “response to ethanol”, “regulation of membrane potential”, and “sodium ion transport”. Within the MF category, 85 terms emerged as significantly enriched, notable among them are “serotonin binding”, “monoamine transmembrane transporter activity”, “monoatomic cation channel activity”, “sodium:chloride symporter activity”, and “monoatomic anion:sodium symporter activity”. For CC, 35 terms were notably enriched, encompassing “postsynaptic membrane”, “synaptic membrane”, “neurotransmitter receptor complex”, “plasma membrane signaling receptor complex”, and “mitochondrial intermembrane space”. The corresponding bar chart is available in Supplementary Figure S1.

The results from the KEGG enrichment analysis are illustrated in Figure 6B, which identified 32 significant signaling pathways. These pathways were ranked in descending order based on their -log10(P) values, with key pathways including “Neuroactive ligand-receptor interaction”, “Cocaine addiction”, “cAMP signaling pathway”, “Pathways in neurodegeneration - multiple diseases”, and “Estrogen signaling pathway”. The “Neuroactive ligand-receptor interaction” pathway was found to be the most significantly enriched. The relevant bar chart is available in Supplementary Figure S2, respectively.

### 6.4 Molecular docking

**FIGURE 7:**
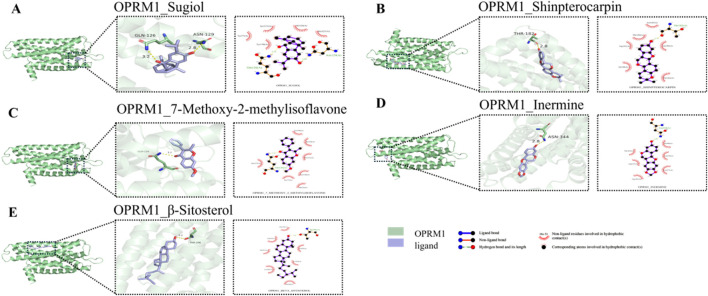
3D and 2D visualization of the top five OPRM1–SPZY ligand interactions from molecular docking analysis. **(A)** OPRM1 and Sugiol; **(B)** OPRM1 and Shinpterocarpin; **(C)** OPRM1 and 7-Methoxy-2-methylisoflavone; **(D)** OPRM1 and Inermine; **(E)** OPRM1 and β-Sitosterol. On the left side of each subfigure: the overall structure of the protein, with the binding pocket of the protein magnified to show details. In the middle: an enlarged view, showing how the small molecule binds to the active site of the protein, with hydrogen bonds and other non-covalent interactions highlighted. Hydrogen bonds are indicated by yellow dashed lines, with the lengths marked around the dashed lines. On the right: A two-dimensional diagram illustrating the types of interactions between proteins and small molecules, such as hydrogen bonds and hydrophobic interactions. The subgraphs are arranged in descending order of binding energy. A to E correspond to ligands Sugiol (ΔG = −8.7 kcal/mol) to β-Sitosterol (ΔG = −7.4 kcal/mol).

**FIGURE 8:**
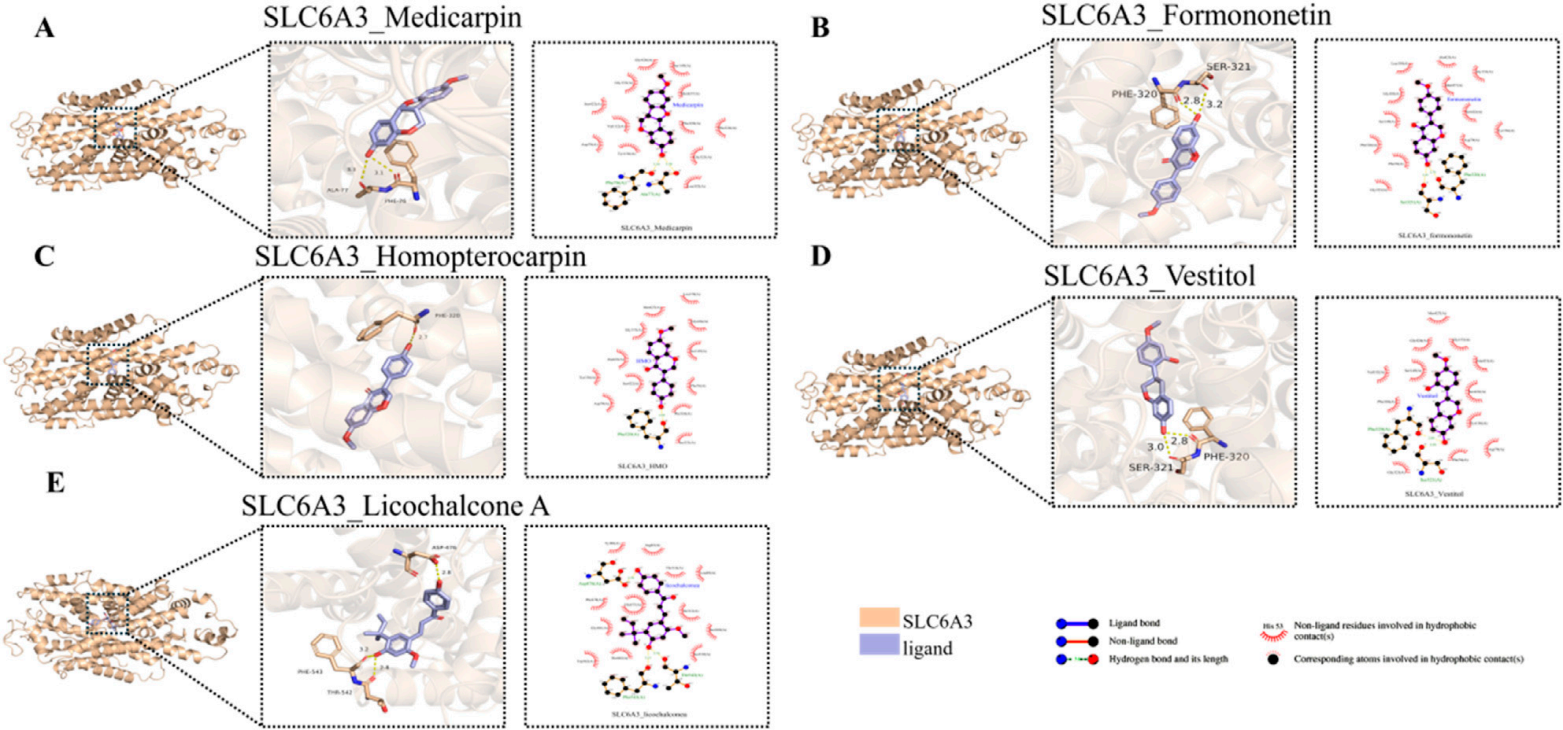
3D and 2D visualization of the top five SLC6A3 –SPZY ligand interactions from molecular docking analysis. **(A)** SLC6A3 and Medicarpin; **(B)** SLC6A3 and Formononetin; **(C)** SLC6A3 and Homopterocarpin; **(D)** SLC6A3 and Vestitol; **(E)** SLC6A3 and Licochalcone **(A)** On the left side of each subfigure: the overall structure of the protein, with the binding pocket of the protein magnified to show details. In the middle: an enlarged view, showing how the small molecule binds to the active site of the protein, with hydrogen bonds and other non-covalent interactions highlighted. Hydrogen bonds are indicated by yellow dashed lines, with the lengths marked around the dashed lines. On the right: A two-dimensional diagram illustrating the types of interactions between proteins and small molecules, such as hydrogen bonds and hydrophobic interactions. A→E arranged in descending order of binding energy, The ligands corresponding to A→E are Medicarpin (ΔG = −9.6 kcal/mol) → Licochalcone A (ΔG = −8.4 kcal/mol).

Figures 7, 8 display the results of the molecular docking analysis, specifically highlighting the five SPZY ligands with the strongest binding energies to the OPRM1 and SLC6A3 receptors, respectively. Sugiol exhibited the highest binding affinity to OPRM1 with a ΔG value of −8.7 kcal/mol, followed by Shinpterocarpin with a ΔG of −7.7 kcal/mol. For SLC6A3, Medicarpin demonstrated the strongest binding with a ΔG of −9.6 kcal/mol, succeeded by Formononetin, which showed a ΔG of −9.4 kcal/mol. Detailed data on these interactions are provided in Table 2.

**TABLE 2:** Results of molecular docking binding energy.

Receptor	Ligand	Binding Energy (cal/mol)
OPRM1	Sugiol	−8.7
	Shinpterocarpin	−7.7
	7-Methoxy-2-methylisoflavone	−7.5
	Inermine	−7.5
	β-Sitosterol	−7.4
SLC6A3	Medicarpin	−9.6
	Formononetin	−9.4
	Homopterocarpin	−9.1
	Vestitol	−9.1
	Licochalcone A	−8.4

Note: This table presents the top five SPZY-derived ligands with the strongest binding affinities (expressed as binding free energy, ΔG, in kcal/mol) to the key targets OPRM1 and SLC6A3, as identified in network pharmacology analysis. Ligands are ranked in descending order of binding strength (most negative ΔG, value first). Lower (more negative) ΔG, values indicate stronger binding stability. Binding energies were calculated using AutoDock Vina.

## 7 Discussion

The principal findings of this study are as follows: 1. Clinical research demonstrates that SPZY effectively and sustainably alleviates symptoms of constipation and depression, thereby enhancing the quality of life for affected individuals. 2. Mass spectrometry analysis suggests that Nobiletin, Tangeritin, and Magnolol may represent novel core active constituents of SPZY. 3. Network pharmacology has identified two primary targets of SPZY: SLC6A3 and OPRM1. 4. Analyses via GO and KEGG indicate significant roles for response to alcohol, regulation of developmental growth, regulation of membrane potential, and neuroactive ligand-receptor interaction in the comorbidity of FC and depression. 5. Molecular docking results reveal that OPRM1 exhibits the highest binding affinity with Sugiol (the principal component of Scrophularia ningpoensis) (ΔG = −8.7 kcal/mol); SLC6A3 displays the highest binding affinity with Medicarpin (the principal component of Astragalus membranaceus) (ΔG = −9.6 kcal/mol).

Mass spectrometry analysis suggests that Nobiletin, Tangeretin, and Magnolol may be the primary active compounds in the SPZY formula. Nobiletin, a citrus flavonoid derived from citrus plants, has been shown to reshape the intestinal microbiota, increase the concentration of short-chain fatty acids in mouse feces, and alleviate symptoms of depression associated with constipation (Kong et al., 2024). Moreover, Nobiletin appears to enhance the number of fecal particles and water content, improve intestinal propulsion rates, and ameliorate colonic histopathological conditions in animal models of constipation. *In vitro* studies have demonstrated that Nobiletin can reverse the increased proliferation of interstitial cells of Cajal (ICC) isolated from constipation models (Zhou et al., 2024). Tangeretin has been shown to effectively improve cognitive impairment (Fatima and Siddique, 2019), and Nobiletin has been proven to possess neuroprotective effects (Braidy et al., 2017). The synergistic anti-neuroinflammatory effects of Nobiletin and Tangeretin are also noted (Ho and Kuo, 2014). Magnolol is renowned for its substantial neuroprotective properties (Sultana et al., 2025). Honokiol can mediate its antidepressant effect through its anti-inflammatory action (Zhang et al., 2019). Notably, key bioactive compounds (e.g., Medicarpin, Sugiol) identified via molecular docking were not among the 50 chromatographically prominent peaks annotated in LC-MS. This may reflect their lower abundance in SPZY or differential ionization efficiency, underscoring that bioactivity is not exclusively correlated with abundance. Their high binding affinity to targets (SLC6A3: ΔG = −9.6 kcal/mol; OPRM1: ΔG = −8.7 kcal/mol) highlights the importance of structure-based activity screening beyond chromatographic detection.

Network pharmacology analysis suggests that SLC6A3 and OPRM1 may be key targets for treating FC combined with depressive symptoms. SLC6A3 encodes the dopamine transporter, responsible for the reuptake of dopamine in the synaptic cleft, directly influencing dopaminergic signal transduction. It has been established that the 9-repeat allele (9R) of SLC6A3 is associated with various mental disorders, including major depressive disorder (Dong et al., 2009; Gontijo et al., 2023), suicidal behavior (Rafikova et al., 2021), and schizophrenia (Sáiz et al., 2010). The expression of SLC6A3 is elevated in animals exhibiting depressive behaviors (Galyamina et al., 2018). The μ-opioid receptor OPRM1 is a primary target of both endogenous and exogenous opioid analgesics. Significant individual variations in human responses to pain stimuli and opioid medications are attributed to genetic variations within the OPRM1 gene (Shabalina et al., 2008). Re-expression of OPRM1 in mouse models has been used to treat pain associated with cancer and to prevent opioid tolerance (Viet et al., 2017). Additionally, polymorphisms in the OPRM1 gene may influence the manifestation of functional gastrointestinal disorders (Adam et al., 2007). Weakened dopaminergic signaling (due to abnormalities in SLC6A3) and dysregulated opioid signaling (stemming from OPRM1 variation) may disrupt the central emotional circuit and intestinal motility regulation through imbalances of neurotransmitters such as GABA and 5-HT. However, current research on SLC6A3 predominantly relates to mental disorders, while studies on OPRM1 primarily focus on pain management. The correlation between FC and these genes remains underexplored and represents a potential avenue for future research.

The relationship between depression, constipation, and the most enriched biological processes - ‘response to alcohol,’ ‘regulation of developmental growth,’ and ‘regulation of membrane potential’ - identified in our GO and KEGG analysis can be interpreted through interconnected neurobiological, gut-brain axis, and molecular pathways. Oxidative stress, a key contributor to both depression and gastrointestinal dysfunction, is exacerbated by chronic alcohol exposure through depletion of antioxidant defenses (e.g., glutathione, GPX, GST), and is frequently elevated in patients with depression (Tsermpini et al., 2022; Liu et al., 2018). Oxidative damage also compromises intestinal barrier function and motility, contributing to constipation (Zeng et al., 2024). Disruptions in developmental growth pathways may impair both intestinal and neural homeostasis. Abnormal proliferation of interstitial cells of Cajal (ICCs) and neural progenitor cells can affect gut motility and neurotransmitter synthesis, respectively, linking developmental alterations to both constipation and depression (Cui et al., 2024; Tu et al., 2024; Zheng et al., 2021)).

Regulation of membrane potential, particularly through ion channels, is crucial for both enteric and central nervous system function. Ion channel dysfunction—such as in potassium, calcium, and sodium channels—may lead to intestinal dysmotility and altered neurotransmission, thereby contributing to mood disturbances (Currò, 2016; Zhao et al., 2023; W. Zhou et al., 2019). Additionally, neurotransmitters like 5-HT, dopamine, and norepinephrine serve dual roles in gut and brain function. Dysregulation of these systems is commonly observed in both depressive states and gastrointestinal disorders, offering a plausible mechanistic link for SPZY’s bidirectional therapeutic effects (Najjar et al., 2023; Mata-Bermudez et al., 2024).

In summary, while these enriched pathways may initially appear peripheral, they provide a biologically plausible framework connecting gut dysfunction and depression. However, further experimental validation is warranted to clarify their roles in SPZY’s mechanisms of action.

The molecular docking studies have elucidated potential interactions between the compounds of the SPZY formula and its two key targets in FC comorbid with depression, including OPRM1 and SLC6A3. OPRM1 demonstrated optimal binding affinity with Sugiol, isolated from Scrophularia ningpoensis (Bajpai et al., 2021) (Chemical Subclass: Diterpenoid). Sugiol is noted for its diverse pharmacological properties, including antibacterial, antioxidant, anti-inflammatory, anticancer, antiviral, and cardiovascular protective activities. To date, there have been no significant updates regarding the pharmacological advancements of Sugiol (Bajpai et al., 2021). *In vitro* studies have indicated that Sugiol’s interaction with STAT3 potentially inhibits the growth and proliferation of gastric cancer cells (SNU-5) (Bakhsh et al., 2023). Conversely, SLC6A3 exhibited the strongest binding energy with Medicarpin, which is predominantly found in Astragalus (Gao et al., 2024) and Glycyrrhiza (Lu et al., 2023) (Chemical Subclass: Pterocarpan). Recent discoveries have shown that Medicarpin mitigates hypoxia-reoxygenation-induced damage in brain microvascular endothelial cells by modulating the PI3K/Akt/FoxO pathway. Additionally, Medicarpin offers protective benefits against cerebral ischemia-reperfusion injury (Oh et al., 2022), has antioxidant properties (Kim et al., 2022), and enhances bone health (Tyagi et al., 2010). Formononetin, recognized for its extensive pharmacological effects in neurological disorders, organ damage, and cancer (Min Jin et al., 2025; M. Jin et al., 2025), has been shown to ameliorate high glucose-induced endothelial dysfunction and inhibit the JAK/STAT signaling pathway (Zhou et al., 2019). However, the associations between these components and OPRM1, SLC6A3, and their therapeutic mechanisms for treating constipation and depression are reported here for the first time.

Study Limitations: Firstly, the absence of a placebo control group presents a limitation. Given that all participants were outpatients with chronic constipation, who had developed resistance to over-the-counter laxatives, addressing their urgent treatment needs posed practical challenges in conducting a randomized controlled trial (RCT). Establishing a placebo control could have raised ethical concerns, such as treatment delays, potentially resulting in poor patient compliance. Although adopting a single-arm study design and mitigating bias risk by enlarging the sample size (N = 202) and enforcing stringent quality control measures. Consequently, our findings should be interpreted as preliminary evidence for SPZY’s efficacy. Future randomized trials with active controls (e.g., osmotic laxatives) are warranted to establish causal relationships. Secondly, while mass spectrometry effectively analyzes the chemical constituents of SPZY (such as Nobiletin, Tangeritin, Magnolol), it does not directly reveal the pharmacokinetic characteristics (e.g., bioavailability, tissue distribution) of these components in humans or their dynamic interactions with targets. Future research should integrate both *in vitro* and *in vivo* experiments to validate their biological activities. Thirdly, the key targets (SLC6A3, OPRM1) and the top 10 ligands identified in molecular docking identified through network pharmacology and molecular docking have yet to be confirmed by functional experiments. Currently, our team has initiated animal studies aimed at systematically analyzing the “component-target-pathway” mechanisms of SPZY by measuring blood concentrations, tissue distributions, and target regulation effects of the identified components.

## 8 Conclusion

The proposed mechanisms underlying the efficacy of SPZY in treating FC combined with depressive symptoms may encompass a variety of biological processes. These include the response to alcohol, the regulation of developmental growth, the modulation of membrane potential, and neuroactive ligand-receptor signaling. Key potential targets implicated in the actions of SPZY are SLC6A3 and OPRM1. It is hypothesized that SPZY’s dual therapeutic effects on FC coupled with depressive symptoms are mediated by the binding of the top 10 ligands identified in molecular docking. To substantiate these mechanisms, further *in vivo* and *in vitro* studies are essential.

## Data Availability

The original contributions presented in the study are included in the article/Supplementary Material, further inquiries can be directed to the corresponding authors.

## Abbreviations

SPZY, Shi Pi Zeng Ye formula; FC, Functional Constipation; HAMD, Hamilton Depression Rating Scale; WCS, Wexner Constipation Assessment Scale; PAC-QOL, Patient Assessment of Constipation Quality of Life; HAMA, Hamilton Anxiety Rating Scale; TCM, Traditional Chinese Medicine; UHPLC, Ultra-high Performance Liquid Chromatography; ESI, Electrospray Ionization; MS_2_, Tandem Mass Spectrometry; PPI, Protein-Protein Interaction; GO, Gene Ontology; KEGG, Kyoto Encyclopedia of Genes and Genomics; PDB, Protein Data Bank; ΔG, Binding Free Energy. AKT1, RAC-alpha serine/threonine-protein kinase; SOD1, Superoxide dismutase [Cu-Zn]; SLC6A, Sodium-dependent dopamine transporter; POMC, Pro-opiomelanocortin; IL6, Interleukin-6; OPRM1, Mu-type opioid receptor; BDNF, Neurotrophic factor BDNF precursor form; GRIA1, Glutamate receptor 1; SLC6A4, Sodium-dependent serotonin transporter; HTR3A, 5-hydroxytryptamine receptor 3A; GRIN1, G protein-regulated inducer of neurite outgrowth 1.
