# Supplementary material for: Clinical efficacy and regulatory mechanisms of Shi Pi Zeng Ye formula in treating functional constipation comorbid with depression: integrating clinical observation, mass spectrometry, bioinformatics, and molecular docking
Source: Front Pharmacol. 2025 Aug 20;16:1645277. doi: 10.3389/fphar.2025.1645277 (PMC12404943; doi:10.3389/fphar.2025.1645277)
Supplement: Supplementary file 1 [file DataSheet2.pdf]

Hamilton Depression Scale (HAMD-17 items)

Developed by Hamilton in 1960.

(1) Depressive mood 0= asymptomatic; 1= Only describe when asked; 2= Express spontaneously in conversation; 3= This emotion can be expressed through expressions, postures, voices, or the urge to cry without words; 4= The patient's verbal and non-verbal expressions (expressions, movements) are almost entirely expressed as this emotion.

(2) Guilt 0= Asymptomatic; 1= Blaming oneself, feeling that one has caused trouble for others; 2= Thinking that you have committed a crime, or thinking over past mistakes or errors; 3= Thinking that the current illness is a punishment for one's own mistakes, or having delusions of sin; 4= Delusions of guilt accompanied by hallucinations of accusation or threat.

(3) Suicide 0= asymptomatic: 1= feeling that life is meaningless; 2= wishing you were dead, or constantly thinking about things related to death; 3= Negative thoughts (suicidal thoughts); 4= Having serious suicidal behavior.

(4) Difficulty falling asleep 0= Asymptomatic; 1= complaining of difficulty falling asleep, that is, still unable to fall asleep half an hour after going to bed; 2= Complaining of difficulty falling asleep every night.

(5) Light sleep 0= Asymptomatic; 1= Light sleep, frequent nightmares; 2=

Waking up in the middle of the night (before midnight) (excluding going to the toilet).

(6) Early awakening 0= asymptomatic; 1= Early awakening, waking up 1 hour earlier than usual, but being able to fall back asleep; 2= Unable to fall back asleep after waking up early.

(7) Work and interest 0= Asymptomatic; 1= narrated when asked; 2= Expressing spontaneously, directly or indirectly, a loss of interest in activity, work, or study, such as feeling listless, indecisive, unable to persist, or having to force oneself to work or activity; 3= Reduced activity time or decreased efficiency, inpatients participating in inpatient work or recreation for less than 3 hours a day; 4= Stop working due to the current illness, the inpatient does not participate in any activities or is unable to complete the daily tasks of the ward without the help of others.

(8) Retardation (slow thinking and speech, difficulty concentrating, and reduced initiative) 0= asymptomatic; 1= Mild retardation found in a mental examination; 2= Obvious sluggishness found in mental examination; 3= Difficulty in conducting a mental examination; 4= Completely unable to answer questions (catatonia).

(9) Agitation 0= asymptomatic: 1= Appearing somewhat distracted during the examination; 2= Obvious restlessness or fidgeting; 3= Inability to sit still, having stood up during the examination; 4= Rubbing hands, biting fingers, pulling hair,

biting lips.

(10) Mental anxiety 0= asymptomatic; 1= Ask for prompt narration; 2= Express spontaneously; 3= Expressions and words showing obvious anxiety; 4= Obvious panic.

(11) Somatic anxiety (referring to the physical symptoms of anxiety, including dry mouth, bloating, diarrhea, belching, colic, palpitations, headache, hyperventilation and sighing, as well as frequent urination and sweating, etc.) 0= asymptomatic; 1= Mild; 2= Moderate, with definite above symptoms; 3= Severe, the above symptoms are serious, affecting life and requiring treatment; 4= Seriously affecting life and activity.

(12) Gastrointestinal symptoms 0= asymptomatic; 1= Loss of appetite, but eating without the encouragement of others; 2= Need to be urged or requested by others to eat and need laxatives or digestive AIDS.

(13) General symptoms 0= asymptomatic; 1= A feeling of heaviness in the limbs, back or neck, back pain, headache, muscle pain, general weakness or fatigue; 2= Obvious symptoms.

(14) Sexual symptoms (referring to decreased libido, menstrual disorders, etc.) 0= no symptoms; 1= mild; 2= severe; 9= Not certain, or the item is not suitable for the person being evaluated (not included in the total score).

(15) Hypochondria 0= asymptomatic; 1= excessive concern for the body; 2= Repeatedly thinking about health issues; 3= Having hypochondriacal delusions; 4= Hypochondriacal delusions with hallucinations.

(16) Weight loss

A assessed by medical history:

0= Asymptomatic; 1= The patient describes possible weight loss; 2= Definite weight loss;

Doctor B measures weight:

0= Weight record indicates a loss of less than 0.5 kilograms in one week; 1= Weight record shows weight loss of more than 0.5 kilograms in one week; 2= Weight record shows weight loss of more than 1 kilogram in one week.

(17) Self-awareness 0= knowing that one is ill, manifested as depression; 1= Knowing that you are ill but blaming it on poor food, environmental problems, being too busy with work, viral infections, the need for rest, etc. 2= Completely deny being ill.

Result analysis:

Mild depression: HAMD 17-item score >7, ≤17;

Moderate depression: HAMD 17-item score >17, ≤ 24;

Severe depression: HAMD 17-item score >24.
