# Supplementary material for: Clinical efficacy and regulatory mechanisms of Shi Pi Zeng Ye formula in treating functional constipation comorbid with depression: integrating clinical observation, mass spectrometry, bioinformatics, and molecular docking
Source: Front Pharmacol. 2025 Aug 20;16:1645277. doi: 10.3389/fphar.2025.1645277 (PMC12404943; doi:10.3389/fphar.2025.1645277)
Supplement: Supplementary file 3 [file DataSheet4.pdf]

Hamilton Anxiety Scale

(Hamilton Anxiety Scale, HAMA)

Instructions for filling out the form: To be completed by trained professionals. Select the most appropriate option under the corresponding choices. Only one option can be chosen for each question.

The scoring criteria:

0 points: Asymptomatic.

1 point: Mild symptoms.

2 points: Moderate symptoms (definite symptoms present but do not affect daily life and activities).

3 points: Severe symptoms (severe symptoms requiring additional treatment or already affecting daily activities).

4 points: Severe symptoms (extremely severe symptoms, seriously affecting their life)

Project	Evaluation criteria				
	Asymptomatic: 0 points	Symptoms are 1 point milder.	Moderate symptoms: 2 points	Symptoms are severe: 3 points.	Symptoms are extremely severe: 4 points
Anxiety: Feeling worried and concerned, anticipating the worst to happen, and being easily irritated.	☒	☒	☒	☒	☒
2. Tension, a sense of tension, easy fatigue, inability to relax, emotional reactions, prone to crying, trembling, and feeling uneasy.	☒	☒	☒	☒	☒
3. Fear<br>Fear of darkness, strangers, being alone, animals, traveling by vehicle or during trips, and crowded places.	☒	☒	☒	☒	☒
4. Insomnia: Difficulty falling asleep, easy to wake up, light sleep, frequent dreams, nightmares, night terrors, and feeling tired after waking up.	☒	☒	☒	☒	☒

5. Cognitive function, also known as memory and attention disorders. Inability to concentrate and poor memory.	☒	☒	☒	☒	☒
6. Depressed mood: Loss of interest, lack of pleasure in previous hobbies, melancholy, early awakening, with symptoms being more severe during the day and less at night.	☒	☒	☒	☒	☒
7. Symptoms of the Muscular System: Muscle soreness, inflexibility in movement, muscle twitching, limb twitching, chattering teeth, and trembling voice.	☒	☒	☒	☒	☒
8. Symptoms of the sensory system: Blurred vision, chills and fever, weakness, and a tingling sensation throughout the body.	☒	☒	☒	☒	☒
9. Cardiovascular system symptoms: Tachycardia, palpitations, chest pain, throbbing sensation in blood vessels, feeling of fainting, and dropped beats.	☒	☒	☒	☒	☒
10. Respiratory system symptoms: Chest tightness, suffocation, sighing, shortness of breath.	☒	☒	☒	☒	☒
11. Gastrointestinal symptoms: Difficulty in swallowing, belching, indigestion (abdominal pain after eating, burning pain in the stomach, abdominal distension, nausea, early satiety), intestinal motility, rumbling in the intestines, diarrhea, weight loss, constipation.	☒	☒	☒	☒	☒
12. Genitourinary system symptoms: frequent urination, urgency, amenorrhea, frigidity, premature ejaculation, impotence, erectile dysfunction.	☒	☒	☒	☒	☒
13. Autonomic nervous system symptoms: dry mouth, flushing, paleness, excessive sweating, goose bumps, tension headache, and hair standing on end.	☒	☒	☒	☒	☒

14. Behavior during the meeting<br>General manifestations: Nervousness, inability to relax<br>be relaxed, feel uneasy, bite one's fingers<br>clenching fists tightly, fiddling with handkerchiefs, facial muscles<br>Twitching, restlessness, stamping of feet, trembling of hands<br>Frowning, stiff expression, high muscle tone<br>Sighing breaths and pale complexion.<br>(2) Physiological manifestations: Swallowing, sneezing<br>When at rest, my heart rate and breathing are fast.<br>Hyperactive tendon reflexes (more than 20 times per minute)<br>In, tremor, dilated pupils, eyelids<br>Palpitations, excessive sweating, and bulging eyes.	⊠	⊠	⊠	⊠	⊠
Total score					
Factor score					
Somatic anxiety (items 7-13)					
(2) Mental anxiety (Items 1-6, 14)					

Introduction

The Hamilton Anxiety Scale (HAMA), developed by Hamilton in 1959, is one of the commonly used scales in clinical psychiatry. It is particularly suitable for assessing the severity of anxiety symptoms but is not often used to evaluate the anxiety state in various mental disorders. HAMA is applicable to adults with anxiety symptoms, especially those with anxiety neurosis. Since neurotic patients often have both depression and anxiety, and the items of HAMA and HAMD overlap, it is difficult to make a distinction based on them.

Notes on Evaluation

The assessor rates the patient by combining conversation and observation according to the content of the scale. It takes 10 to 15 minutes to complete one assessment.

The time frame for assessment: Generally, the current situation or the most recent week is evaluated. The assessment interval can also be set as needed, such as re-assessing 2 to 6 weeks after treatment to compare the changes in symptoms and conditions before and after treatment.

All items are scored based on the patient's oral account, except for item 14, which requires a combination of observation. At the same time,

Particular emphasis is placed on the subjective experience of the examinee, which is also the medical perspective of the HAMA developers. Since patients only seek medical attention and treatment when they have a subjective feeling of illness, this can be used as a criterion for the severity of the condition and whether there has been improvement.

Result Analysis

1 Total score

It can well reflect the severity of the condition. The scale collaboration group once compared the total scores of HAMA of 230 patients with different subtypes of neurosis. The average total score of neurasthenia was 21.00, that of anxiety disorder was 29.25, and that of depressive neurosis was 23.87. Therefore, anxiety symptoms are the prominent manifestation in patients with anxiety disorder. This group of patients was a set of anxiety disorder patients with relatively severe conditions.

2Factor scores

The HAMA is only divided into two major factor structures: somatic and psychic.

(1) Somatic anxiety: It consists of seven items, namely, symptoms of the muscular system, sensory system, cardiovascular system, respiratory system, gastrointestinal system, genitourinary system, and autonomic nervous system.

(2) Mental anxiety: Composed of the remaining 7 items: items 1, 2, 3, 4, 5, 6 and 14. Through factor analysis, it can not only specifically reflect the mental pathology characteristics of the patient, but also reflect the treatment outcome of the target symptom cluster.

The total score is the sum of all items, ranging from 0 to 56. According to the data provided by the National Scale Collaboration Group, a total score exceeding 29 indicates severe anxiety; 29 to 22 suggests significant anxiety; 21 to 15 indicates definite anxiety; 14 to 7 implies possible anxiety; and a score below 7 indicates no anxiety. Generally, a total score of 14 on the 14-item HAMA is used as the cut-off value.

App Review

The wide application of HAMA has demonstrated its excellent quality and broad applicability. It is mostly used in the assessment of anxiety symptoms. HAMA can be used alone to evaluate the level of anxiety or in combination with specific anxiety symptom scales to comprehensively consider the general anxiety level and the severity of specific symptoms. It has a relatively good consistency. The assessment takes 15 to 20 minutes, which is of moderate length and easy to conduct.

References:

[1]Hamilton M. The assessment of anxiety by rating scale. Brit. J. Med Psychol.1959,

32:50-55

[2]蔺国宪, 等.汉密顿焦虑量表(HAMA)在神经症中的应用.中华神经精神科杂志, 1986,19:342-344
