# Supplementary figures and images for: Clinical efficacy and regulatory mechanisms of Shi Pi Zeng Ye formula in treating functional constipation comorbid with depression: integrating clinical observation, mass spectrometry, bioinformatics, and molecular docking
Source: Front Pharmacol. 2025 Aug 20;16:1645277. doi: 10.3389/fphar.2025.1645277 (PMC12404943; doi:10.3389/fphar.2025.1645277)

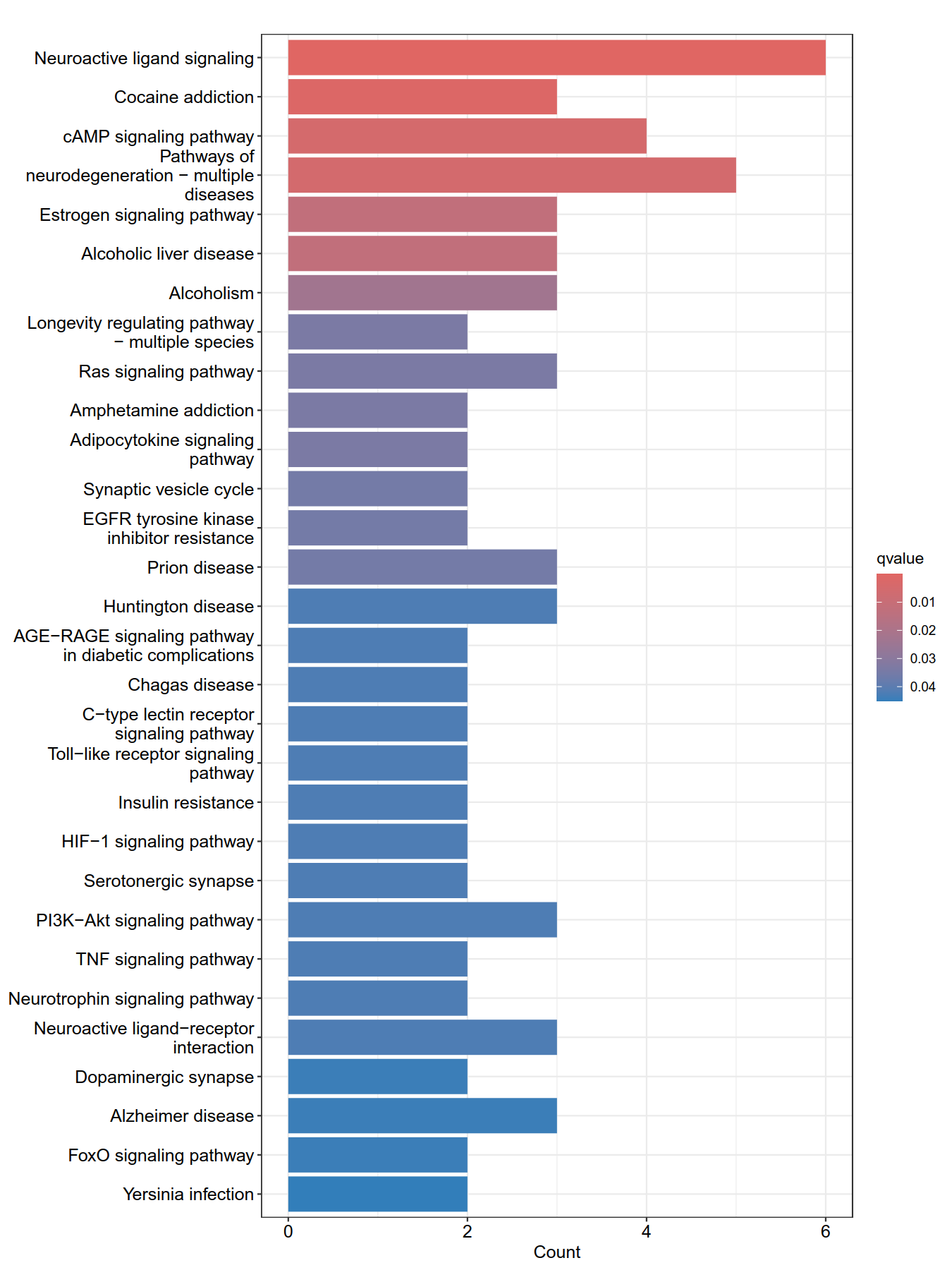

Supplement: Supplementary file 5 [file Image2.tif]

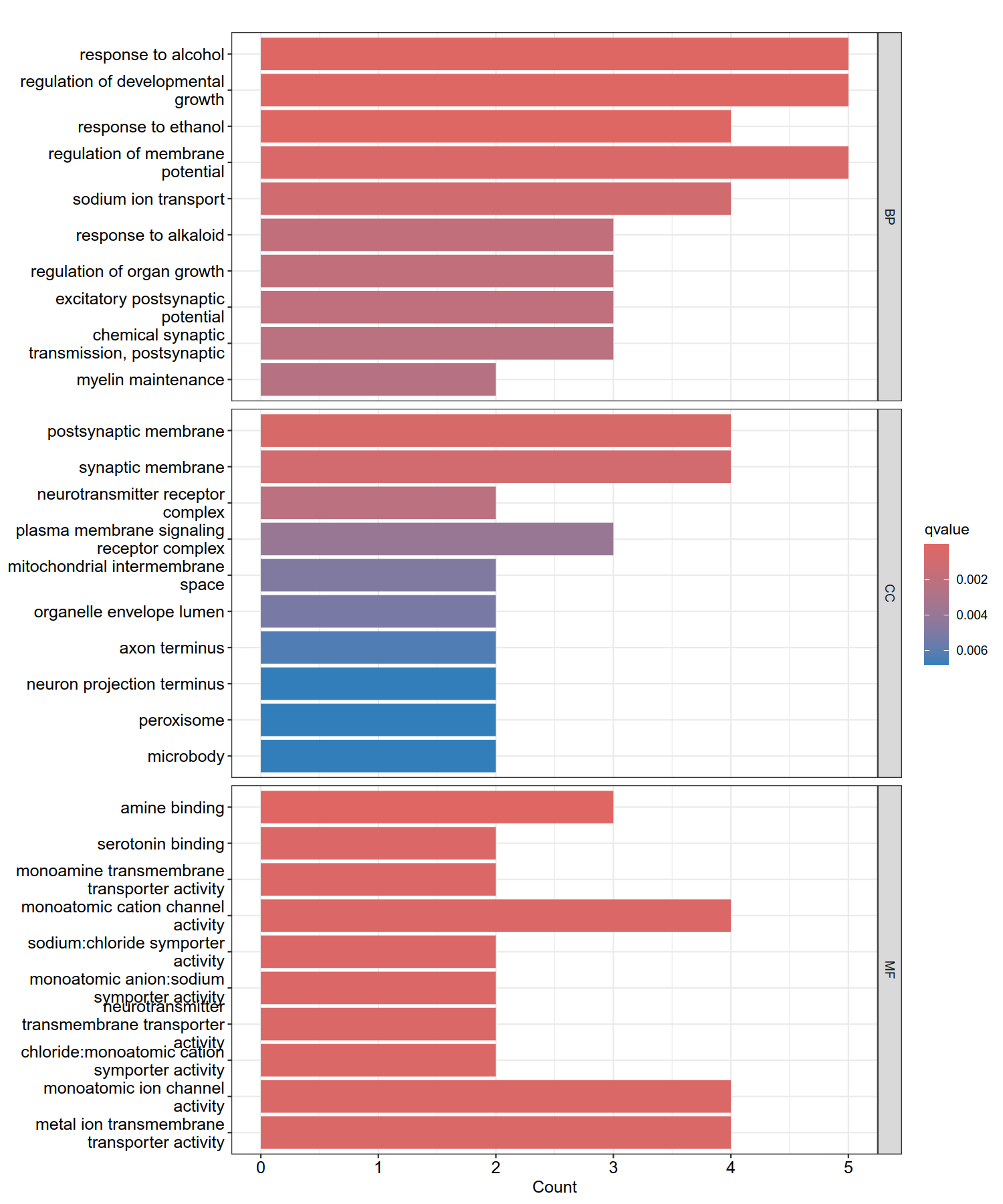

Supplement: Supplementary file 6 [file Image1.tif]
