# Supplementary material for: Clinical efficacy and regulatory mechanisms of Shi Pi Zeng Ye formula in treating functional constipation comorbid with depression: integrating clinical observation, mass spectrometry, bioinformatics, and molecular docking
Source: Front Pharmacol. 2025 Aug 20;16:1645277. doi: 10.3389/fphar.2025.1645277 (PMC12404943; doi:10.3389/fphar.2025.1645277)
Supplement: Supplementary file 7 [file DataSheet3.pdf]

Quality of Life Scale for Constipation Patients (PAC-QOL)

PAC-QOL reflects the impact of constipation on your daily life over the past two weeks. Choose an answer for each question.

The following questions are related to symptoms of constipation. The severity or intensity of the following symptoms in the past two weeks...	Not at all	A little bit	General	More serious	Very serious
	0	1	2	3	4
1. Feeling bloated?	□	□	□	□	□
2 Feel heavy?	□	□	□	□	□

The following questions about constipation and daily life. How much time in the past two weeks...	No time	Occasionally	Sometimes	Most time	Always
	0	1	2	3	4
3. Feeling unwell?	□	□	□	□	□
4 Feel the urge to defecate but have difficulty doing so?	□	□	□	□	□
5. Feeling uncomfortable in the company of others?	□	□	□	□	□
6. Eating less and less because of constipation?	□	□	□	□	□

The following questions about constipation and daily life, the severity and intensity of the following questions in the past two weeks...	Not at all	A little bit	General	More serious	Very serious
	0	1	2	3	4
7. Must be concerned about what to eat	□	□	□	□	□
8. Loss of appetite	□	□	□	□	□
9 worry about not being able to choose food at will (such as at a friend	□	□	□	□	□

’s house)					
10. Feeling uncomfortable for spending too much time in the bathroom when you're out	□	□	□	□	□
11. Feeling uncomfortable going to the bathroom frequently while on the go	□	□	□	□	□
12. Always worried about changing living habits (such as traveling, going out, etc.)	□	□	□	□	□

The following question is related to the feeling of constipation. The time and frequency of the following symptoms occurring in the past two weeks...	No time	Occasionally	Sometimes	Most time	Always
	0	1	2	3	4
13. Feeling irritable	□	□	□	□	□
14. Feeling uneasy	□	□	□	□	□
15. Always troubled	□	□	□	□	□
16. Feeling nervous	□	□	□	□	□
17. Feeling lacking in confidence	□	□	□	□	□
18. Feeling out of control of life	□	□	□	□	□

The following questions are related to the feeling of constipation. The severity	Not at all	A little bit	General	Rather serious	Very serious
	0	1	2	3	4

and intensity of the following problems over the past two weeks...					
19. Worrying about not knowing when to defecate	□	□	□	□	□
20 Worry about not being able to defecate	□	□	□	□	□
21. To be affected by not defecating	□	□	□	□	□

The following questions about constipation and daily life. The time and frequency of the following symptoms over the past two weeks...	No time	Occasionally	Sometimes	Most time	Always
	0	1	2	3	4
22. Worrying that things are getting worse	□	□	□	□	□
23. Feeling that the body is not functioning	□	□	□	□	□
24. Defecate less frequently than expected	□	□	□	□	□

The following question is about satisfaction. The severity and intensity of the following questions over the past two weeks...	Satisfied	Relatively satisfied	Generally	A little dissatisfied	Very dissatisfied
	0	1	2	3	4
25. Are you satisfied with the number of bowel movements?	□	□	□	□	□
26. Are you satisfied with regular bowel movements?	□	□	□	□	□

27. Are you satisfied with the time it takes for things to pass through your gut?	□	□	□	□	□
28. Are you satisfied with your previous treatments?	□	□	□	□	□

PAC-QoL is a self-reported questionnaire used to measure a patient's quality of life. The verified PAC-QoL consists of 28 items and is divided into four subscales: physical discomfort, psychosocial discomfort, worry and concern, and satisfaction. The first three subscales are used to assess the patient's dissatisfaction index, with total scores ranging from 0 to 96(the lower the score, the better the quality of life). The satisfaction subscale consists of four items, with overall scores ranging from 0 to 16, so each patient's self-reported final outcome is defined as poor (0-4), fairly good (5-8), good (9-12), or excellent (13-16).
