# Supplementary material for: Clinical efficacy and regulatory mechanisms of Shi Pi Zeng Ye formula in treating functional constipation comorbid with depression: integrating clinical observation, mass spectrometry, bioinformatics, and molecular docking
Source: Front Pharmacol. 2025 Aug 20;16:1645277. doi: 10.3389/fphar.2025.1645277 (PMC12404943; doi:10.3389/fphar.2025.1645277)
Supplement: Supplementary file 8 [file DataSheet1.pdf]

Wexner Constipation Score (WCS)

Project	Scoring criteria
Frequency of defecation	□ Zero points. Once or twice every one to two days.<br>□ One point. twice a week<br>□ Two points. Once a week<br>□ 3 points Less than once a week<br>□ 4 points Less than once a month
2. Difficulty: Pain assessment	□ Zero points. never<br>□ One point. seldom<br>□ Two points. Sometimes<br>□ 3 points Usually<br>□ 4 points always
3. Completeness: Incomplete sensory assessment	□ Zero points. never<br>□ One point. seldom<br>□ Two points. Sometimes<br>□ 3 points Usually<br>□ 4 points always
4. Pain: Abdominal pain	□ Zero points. never<br>□ One point. seldom<br>□ Two points. Sometimes<br>□ 3 points Usually<br>□ 4 points always
5. Time: Time spent in the toilet (min)	□ Zero points. Less than five<br>□ One point. 5-10<br>□ Two points. 10-20

	□ 3 points 20-30<br>□ 4 points More than 30
6. Auxiliary: Auxiliary form	□ Zero points. There is none.<br>□ One point. Stimulant laxatives<br>□ Two points. Finger assistance or enema
7. Failure: Number of failed attempts to defecate within 24 hours	□ Zero points. no<br>□ One point. 1 to 3 times<br>□ Two points. 3 to 6 times<br>□ 3 points 6 to 9 times<br>□ 4 points More than 9 times
8. Medical history: Duration of constipation (years)	□ Zero points. 0<br>□ One point. 1-5<br>□ Two points. 5-10<br>□ 3 points 10-20<br>□ 4 points More than 20
Total score	

Introduction

The Wexner scoring system strategy was established by American scholar Agachan et al. in 1996. It mainly consists of eight items: frequency of defecation, difficulty, incomplete evacuation, pain, defecation time, assistance in defecation, failed defecation, and medical history. The severity of constipation is evaluated by the Wexner scoring system for constipation, with a total score of 30 points and a minimum of 0 points. The higher the score, the more severe the constipation.

References

[1] 赵建军, 许丽华. 采用Wexner 评分系统的综合护理在脑梗死患者便秘中的应用[J]. 中华现代护理杂志, 2017, 023(034):4395-4397.
