# Supplementary material for: Clinical efficacy and regulatory mechanisms of Shi Pi Zeng Ye formula in treating functional constipation comorbid with depression: integrating clinical observation, mass spectrometry, bioinformatics, and molecular docking
Source: Front Pharmacol. 2025 Aug 20;16:1645277. doi: 10.3389/fphar.2025.1645277 (PMC12404943; doi:10.3389/fphar.2025.1645277)
Supplement: Supplementary file 9 [file DataSheet5.pdf]

APPLIED PROTEIN TECHNOLOGY 中科新生命

High-resolution mass spectrometry of chemical components in traditional Chinese medicine Analysis Report

Project Name: **Component Identification of Changzhou Tongbian Formula**

Commissioned: Xiyuan Hospital, China Academy of Chinese Medical Sciences

Project Number: YAS202411130039-1

Report Time: **2024-12-04**

Contents

1. Overview 2

2. Project Experiment process 2

3. Laboratory instruments and reagents 3

4. Experimental methods 4

5. Evaluation of experimental data quality 7

6. Experimental results 9

7. References 19

8. Attachments 20

9. Appendix: Methods in Chinese and English (for reference) 20

1. Overview

The chemical composition of traditional Chinese medicine is complex, with a wide variety of structural types, numerous isomers, large differences in component content, and a wide range of polarity. The systematic identification of chemical components in traditional Chinese medicine is conducive to clarifying the material basis of the efficacy of traditional Chinese medicine and the pharmacological effects that exert its efficacy, and plays an important role in the modernization research and quality control of traditional Chinese medicine. Ultra-high performance liquid chromatography (UPLC) - High Resolution Mass Spectrometry (HRMS) combined analysis techniques, which are characterized by rapidity, efficiency, high sensitivity and good selectivity, are increasingly being used in the study of chemical components of traditional Chinese medicine. UPLC, with its large sample loading capacity, good applicability and high resolution, has become an effective means of drug analysis. HRMS can provide high-resolution mass spectra of all ions, first-order precise mass and rich second-order fragment information, with high sensitivity, high resolution, good accuracy and wide detection dynamic range; It can provide high-quality structural information data when detecting complex components of traditional Chinese medicine.

This project uses ultra-high performance liquid chromatography-high resolution mass spectrometry Q-Exactive HFX to detect the chemical components in the samples, and the chemical substances in the samples are structurally identified by matching with the first-level precise mass (molecular mass error <25 ppm) and second-level fragment spectrum information of the compounds in the local standard database.

2. Project Experimental procedures

2.1 Pre-experiment

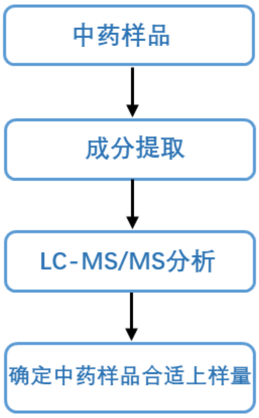

2.2 Formal experiment

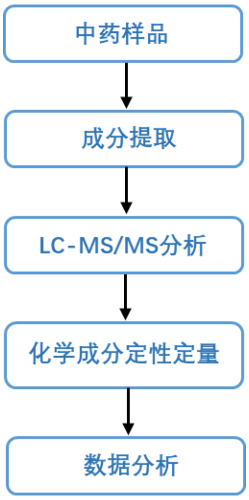

3. Laboratory instruments and reagents

3.1 Instruments

TQ-Exactive HFX mass spectrometer (Thermo Fisher Scientific, Bremen, Germany)

UHPLC vanquish ultra-high performance liquid chromatograph (Thermo Fisher Scientific, Bremen, Germany)

Low-temperature high-speed Centrifuge: Eppendorf Centrifuge 5430 R(Eppendorf, Hamburg, Germany)

Electronic balance: Mettler Toledo, ME104 (Mettler Toledo, Shanghai, China)

Vortex: Xiexu Medical Technology, QT-1 (Xiexu Medical, Shanghai, China)

Freeze dryer: FD-1C-80 (Shanghai Bilon instruments Co.,Ltd., Shanghai, China)

Ultrasonic cleaner: SB-4200D (Ningbo Scientz Biotechnology Co.,Ltd., Ningbo, China)

3.2 reagent

The information of reagents and pure solvents used in this project is shown in Table 1.

Table 1 Solvent and Pure Solvent Information

Names	Supplier	Level
water	Millipore	Mass spectrometry level
Acetonitrile	Fisher-chemical	Mass spectrometry grade
Methanol	Fisher-chemical	Mass spectrometry grade
Formic acid	Honeywell	Mass spectrometry grade

4. Experimental methods

4.1 Sample Information

Sample information to be tested: Specific sample information is shown in Table 2.

Table 2 Sample Information

Sample group name	Sample name abbreviation	Sample quantity
**Changzhou Tongbian Formula**	CZTBF	1

4.2 Preparation of Chinese medicine samples

Take this sample and grind it into powder at room temperature; Accurately weigh 100 mg into a 1.5 mL centrifuge tube; Add 1 mL of 70% methanol to dissolve in water; Fully vortex for 30 seconds; Water bath ultrasound (power 600W, frequency 40kHz) 30 min; Centrifuge for 10 minutes (16000 g, 4 ° C);

Transfer the supernatant to a 96-well protein filter plate under positive pressure nitrogen filtration; Transfer the filtrate to a 2 mL EP tube and vacuum dry; Add 400 μL of 40% methanol aqueous solution on top to redissolve; Vortex to mix for 30 seconds, centrifuge for 10 minutes (16000 g, 4 ° C), and take the supernatant.

4.3 Chromatography-mass spectrometry conditions

4.3.1 Chromatographic conditions

Samples were treated using the Vanquish UHPLC (Thermo Fisher Scientific, Bremen, Germany) ultra-high performance liquid chromatography system combined with ACQUITY UPLC HSS T3 (2.1 mm X 100 mm) Separation was carried out using a 1.8 μm column; Column temperature 35 ° C; Flow rate 0.3 mL/min; Mobile phase composition A 0.1% formic acid aqueous solution B 0.1% formic acid acetonitrile solution; Perform gradient elution as shown in the table below

Time (minutes)	Mobile phase A (%)	Mobile phase B (%)
Initial	95	5
3	75	25
8.5	55	45
14	5	95
17	2	98
17.2	95	5
20.0	95	5

4.3.2 Mass spectrometry conditions

Sample primary and secondary spectra were collected using a Q-Exactive HFX mass spectrometer.

The Q-Exactive HFX mass spectrometer samples were combined with the UHPLC system, and the electrospray ionization (ESI) positive and negative ion modes were used for mass spectrometry collection respectively. Spray voltage 3800

≈ 34 psi ≈ 15 psi

V (ESI+) /3500V (ESI-), sheath gas pressure 45arb, auxiliary gas pressure 20arb, ion transport tube temperature 320 ° C, atomization temperature 350 ° C; The detection mode is full-scan/data-dependent two-stage scan (Full-MS/ dd-MS2), with first-stage

and second-stage resolutions of 60,000 and 15,000 respectively. The top 10 MS1 ions obtain MS/MS spectra, and the collision energy (CEs) uses stepwise normalized energy levels 20, 40, 60; The first-level mass-to-charge ratio scan range is 90 to 1300.

4.3.3 Sample detection and analysis

Accurately pipette 6 μL of CZTBF solution, LC-MS injection analysis, repeat the CZTBF sample injection 5 times.

4.4 Data analysis process

The raw data file in.raw format was imported into proteoWizard for conversion to.mzxml format. Peak alignment, retention time correction and peak extraction were performed using XCMS software. Compound identification was conducted by searching the local Chinese medicine high-resolution mass spectrometry database of Zhongke Xinlife with a first-level mass error of less than 25 ppm. The second-level fragmentation profile match score is greater than 0.7, where the higher the score, the higher the profile similarity. Currently, it[2-3] is generally believed that the identification results are reliable when the score is above[4-5] 0.7.

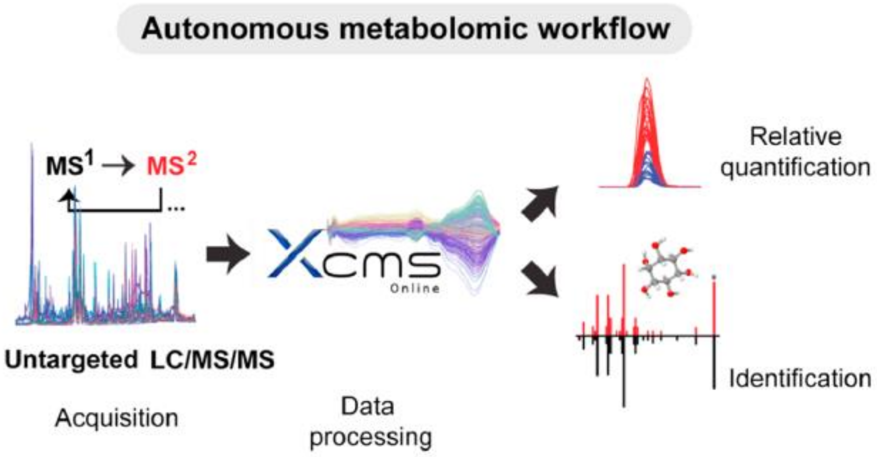

Raw data preprocessing process

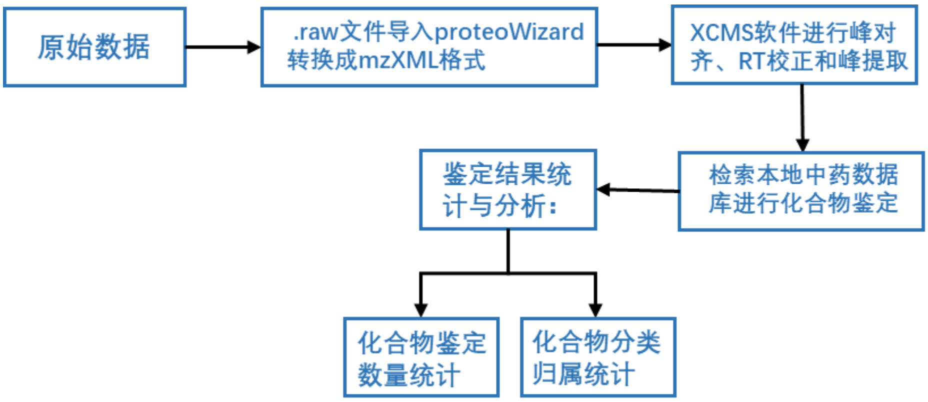

Data analysis process

5. Evaluation of experimental data quality

This experiment evaluated the stability of the instrument and the reliability of the data. Pearson correlation analysis was conducted on the test results of the Chinese herbal samples, as shown in Figures 1a and 1b. Generally, a correlation coefficient greater than 0.9 indicates a good correlation. The results of this experiment show that the correlation coefficients among the samples are all above 0.9, indicating good repeatability of the experiment and stable and reliable data.

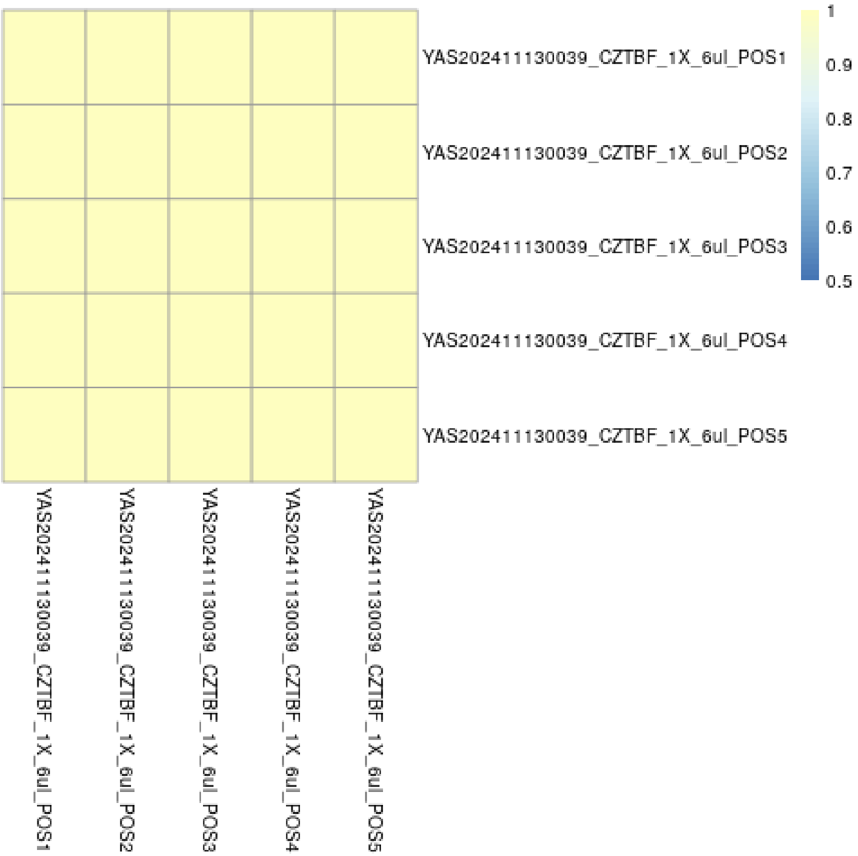

Figure 1a Correlation of Chinese medicine samples in the positive ion mode

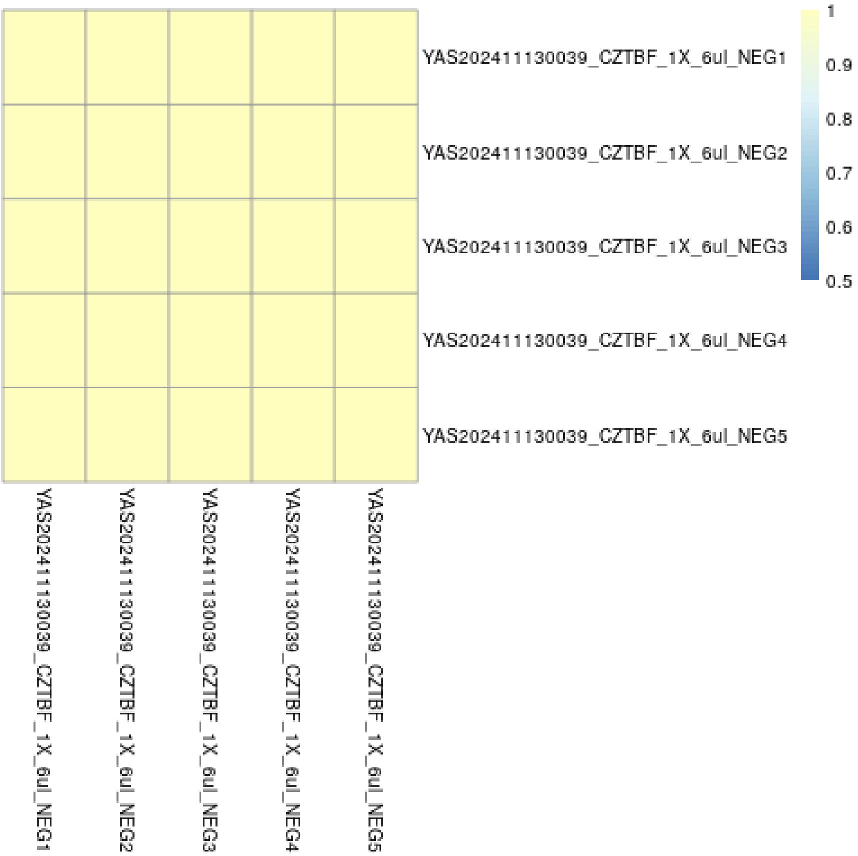

Figure 1b Correlation of Chinese medicine samples in negative ion mode

Note: The horizontal and vertical axes in the figure represent the repeated detection data of traditional Chinese medicine samples. The dots in each small grid represent the ion peaks (compounds) extracted from the Chinese medicine samples, and the horizontal and vertical axes represent the logarithms of the signal intensity values of the ion peaks.

6. Experimental Results

6.1 Compound identification results

6.1.1 Quantity and classification of compounds identified in Chinese medicine samples

Compound identification was conducted by searching the local Chinese me dicine high-resolution mass spectrometry database of Zhongke Xinlife, with a fi rst-level mass error of less than 25 ppm and a second-level fragmentation spectr al matching score of more than 0.7. The chemical components in the identified Chinese medicine samples were analyzed, and the statistical results are shown i n Table 3. The specific names of the chemical components obtained from the id entification of the Chinese medicine samples and related information can be fou nd in **"6_ Attachment: Table 2_ Results of Chemical Component Identificat ion"**.

Table 3 Statistics of the number of compounds Identified in positive and negative ion modes

Detection of ion patterns	Identifying [illegible]
**Positive ion mode (POS)**	1522
**Negative ion mode (NEG)**	930
**Combined positive and negative ion patterns**	2362

Note: Total of positive and negative ion modes: If the same compound is identified in both positive and negative ion modes, it is not counted repeatedly

The compounds identified in this experiment (compounds identified in com bination with positive and negative ions) were annotated according to the NPCl assifier[1] classification method. The statistics of the number of compounds ident ified in each pathway and major superclass are shown in Table 4, and the prop ortion of each compound classification is shown in Figure 3a. For the specific c

Add: 上海市闵行区园美路 58 号 1 号楼 1 building, 58 Yuan-mei Rd. Shanghai China 200233 | Tel: +86 021-5497 5031| E-mail: info_apt@sibs.ac.cn

lassification information of each type of compound, see **"3_ Attachment: Table 1 Compound Categories and Quantities".**

Table 4 Categories and Quantities of Compounds

Pathway	SuperClass	count
Alkaloids	Anthranilic acid alkaloids	38
Alkaloids	Lysine alkaloids	34
Alkaloids	Nicotinic acid alkaloids	37
Alkaloids	Tryptophan alkaloids	65
Alkaloids	Tyrosine alkaloids	53
Alkaloids	other	210
Amino acids and Peptides	Amino acid glycosides	3
Amino acids and Peptides	Oligopeptides	5
Amino acids and Peptides	Small peptides	157
Amino acids and Peptides	other	4
Amino acids and Peptides	Beta lactams	1
Carbohydrates	Aminosugars and aminoglycosides	5
Carbohydrates	Nucleosides	19
Carbohydrates	Polyols	3
Carbohydrates	Saccharides	32
Carbohydrates	other	2
Fatty acids	Fatty Acids and Conjugates	104
Fatty acids	Fatty amides	19
Fatty acids	Fatty esters	14
Fatty acids	Glycerophospholipids	26
Fatty acids	Octadecanoids	32
Fatty acids	other	58
Polyketides	Aromatic polyketides	15
Polyketides	Chromanes	10
Polyketides	Cyclic polyketides	20
Polyketides	Macrolides	10
Polyketides	Polycyclic aromatic polyketides	8
Polyketides	other	45
Shikimates and Phenylpropanoids	Coumarins	50
Shikimates and Phenylpropanoids	Flavonoids	223
Shikimates and Phenylpropanoids	Isoflavonoids	58
Shikimates and Phenylpropanoids	Phenolic acids (C6-C1)	64
Shikimates and Phenylpropanoids	Phenylpropanoids (C6-C3)	70
Shikimates and Phenylpropanoids	other	189

Terpenoids	Diterpenoids	24
Terpenoids	Monoterpenoids	83
Terpenoids	Sesquiterpenoids	115
Terpenoids	Steroids	65
Terpenoids	Triterpenoids	97
Terpenoids	other	39
other	Meroterpenoids	15
other	Phenylpropanoids (C6-C3)	10
other	Pseudoalkaloids	3
other	Small peptides	13
other	Tetramate alkaloids+Peptide alkaloids	2
other	other	33

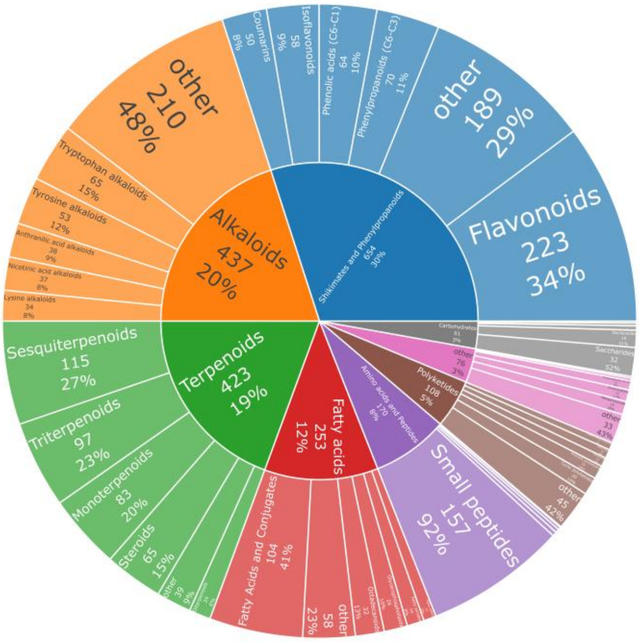

Figure 3a Proportion of Chinese medicinal compounds in each chemical classification

Note: The classification of identified compounds in traditional Chinese medicine shows the top5 categories of pathway and superclass.

NPClassifier Classification description: **NPClassifier** is a structural classification tool based on deep neural networks, specifically designed for natural products (plants, microorganisms, etc.). It automatically classifies natural products (NPS) based on the structural descriptors of compounds -

counting Morgan fingerprints (counted Morgan fingerprints, CMFs) to help understand the molecular structure, chemical properties, biological activity, and biosynthetic pathways of natural products.

The classification system of NPClassifier divides the structure of natural products into three levels: 7 pathways, 70 superclasses and 672 classes.

Among them, the Pathway pathway includes fatty acids, polyketones, shikimate - phenylpropanoids, terpenoids, alkaloids, amino acids/peptides and carbohydrates, which are used to represent different biosynthetic pathways. It should be noted that a natural product can belong to multiple pathways.

A Superclass is a subclass of a Pathway that represents a general category of metabolites (such as flavonoids, terpenoids, or steroids), a general chemical/molecular shape (such as tryptophan, macrolides), or biosynthetic information (such as tryptophan alkaloids, aromatic polyketones, or pseudo-alkaloids).

Classes are further subdivided from superclasses and represent specific compound families (such as erythromycin, penicillin, or cannabinoids), characteristic functional groups (such as chromolones, isoflavones, or indole alkaloids), or skeleton diversity within the superclass (such as flavanones, flavonoids, and chalketones among flavonoids).

6.2.2 Analysis of basal Peak chromatograms (BPC) of traditional Chinese medicine

In the Base peak chromatogram (BPC) of the positive and negative ions of traditional Chinese medicine, peak shape confirmation and secondary chromatogram examination were performed on the chromatographic peaks with higher abundance respectively, and then the positive and negative ion chromatograms were labeled with the peak numbers in numerical order, as shown in Figures 4a and 4b. A total of 50 chromatographic peaks were labeled **in this project. The information of the corresponding identified compounds is shown in Table 5**, and the NPClassifier chemical classification is shown in Figure 5.

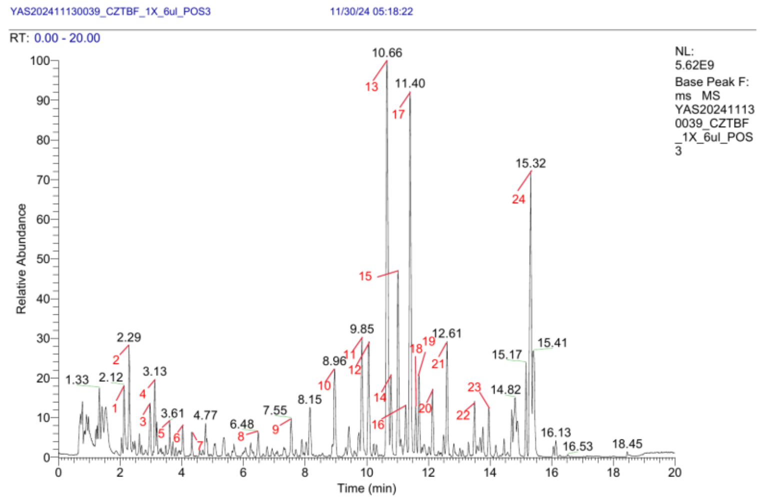

Figure 4a BPC diagram of Chinese medicine (CZTBF) in cation mode - Labeled peaks

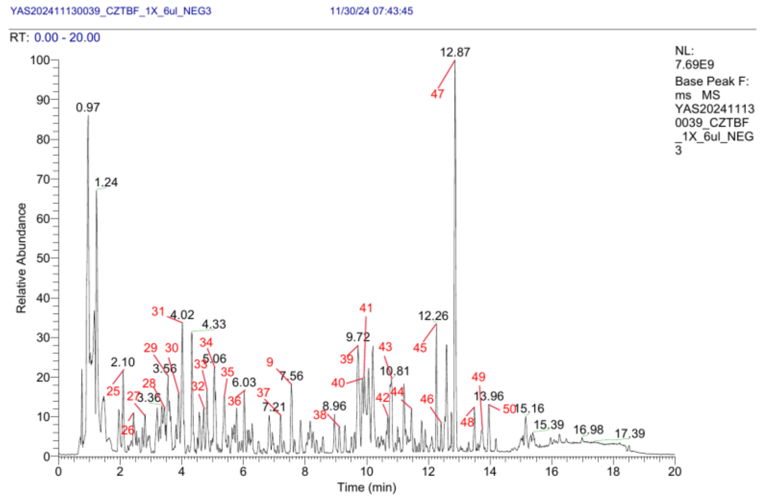

Figure 4b Chinese medicine (CZTBF) BPC pattern in negative ion mode - marked peak

Table 5 Chemical composition identification results of the BPC icon peak

No	m/z	RT/min	ppm	Adduct	score	Compound_EN	Compound_CN	SuperClass
1	145.0496	2.14	1.1	[M+H]+	0.9695	(3E)-3-Hexenedioic acid	Trans-2-butene-1, 4-diformic acid	Fatty Acids and Conjugates
2	166.0857	2.31	0.3	[M+H]+	0.9995	Phenylalanine	Phenylalanine	Small peptides
3	205.0973	2.98	0.2	[M+H]+	0.9747	L-Tryptophan	L-tryptophan	Small peptides
4	314.175	3.14	0.4	[M+H]+	0.9469	Codethyline	Ethyl morphine	Tyrosine alkaloids
5	342.17	3.62	0.1	[M]+	0.9219	Magnoflorine	Magnolia alkine	Tyrosine alkaloids
6	481.1706	4.04	1.3	[M+H]+	0.9949	Albiflorin	Paeoniflorin	Monoterpenoids
7	179.0698	4.33	0.1	[M+H-NH3]+	0.7707	.beta.-Homotyrosine	3-amino-4 -(4-hydroxyphenyl) butyric acid	Small peptides
8	207.1017	6.49	0.3	[M+H-H2O]+	0.9661	6, 7 - Dihydroxyligustilide	6, 7-dihydroxyligustilide	Cyclic polyketides
9	167.0701	7.56	0.2	[M+H-C15H20O8]+	0.9706	Harpagoside	Harpagoside	Monoterpenoids
10	373.1281	8.96	0.3	[M+H]+	0.9995	Isosinensetin	3',4',5,7, 8-pentamethoxysone	Flavonoids
11	373.1281	9.85	0.1	[M+H]+	0.9825	Sinensetin	5,6,7,3',4' -pentamethoxysone	Flavonoids
12	343.1175	10.07	0.8	[M+H]+	0.9156	6-Demethoxytangeretin	6-demethoxytangeretin	Flavonoids
13	403.1387	10.66	0.4	[M+H]+	0.9932	Nobiletin	Nobiletin	Flavonoids
14	343.1175	10.79	0.2	[M+H]+	0.9544	5-Methoxysalvigenin	4',5,6, 7-tetramethoxysalvigenin	Flavonoids

15	433.1493	11.01	0.7	[M+H]+	0.9586	Hibiscetin heptamethyl ether	3',4',5',3,5,7, 8-heptathoxyethanone	Flavonoids
16	419.1338	11.27	0.4	[M+H]+	0.9508	Gardenin	Gardenia (yellow) A	Flavonoids
17	373.1281	11.41	1.8	[M+H]+	0.9901	Tangeritin	Citrusin	Flavonoids
18	231.1381	11.59	0.7	[M+H-CH4O]+	0.9564	8-Methoxyatractylenolide I	NA	Sesquiterpenoids
19	389.1231	11.7	1.4	[M+H]+	0.9941	5-O-Demethylnobiletin	Demethylnobiletin	Flavonoids
20	191.1067	12.13	0.2	[M+H]+	0.9858	Ligustilide A	Artemistilide A	Cyclic polyketides
21	233.1537	12.61	0.5	[M+H]+	0.9686	Atractylenolide II	Atractylenolide II	Sesquiterpenoids
22	520.3401	13.49	0.4	[M+H]+	0.9583	1-Linoleoyl-sn-glycero-3-phosphorylcholine	1-linoleoyl-sn-glycerol-3-phosphorylcholine	Glycerophospholipids
23	496.3402	13.97	0.7	[M+H]+	0.9754	Lyso-PC(16:0)	Palmitoyllysophosphatidylcholine	Glycerophospholipids
24	282.2791	15.32	0.1	[M+H]+	0.8977	Oleamide	Oleamide	Fatty amides
25	391.1244	2.08	0.3	[M+HCO2]-	0.9797	Aucubin	Peach leaf coralline	Monoterpenoids
26	409.1348	2.44	0.9	[M+HCOO]-	0.9159	Harpagide	Harpagide	Monoterpenoids
27	353.0876	2.81	0.8	[M-H-C9H6O3]-	0.9838	Cynarin	Silymarin	Phenylpropanoids (C6-C3)
28	165.0549	3.43	4.7	[M-H]-	0.9973	Dihydro-m-coumaric acid	3-(3-hydroxyphenyl) propionic acid	NA
29	502.1562	3.55	0.3	[M+HCOO]-	0.885	Amygdalin	Amygdalin	Amino acid glycosides
30	165.0185	3.9	4.7	[M-H]-	0.9984	4-Formyl-2-hydroxybenzoic acid	4-formyl-2-hydroxybenzoic acid	Phenolic acids (C6-C1)
31	167.0341	4.02	5.0	[M-H]-	0.999	3, 4-dihydroxyphenylacetic acid	3, 4-dihydroxyphenylacetic acid	Phenolic acids (C6-C1)
32	549.1611	4.69	1.3	[M-H]-	0.9617	Liguiritigenin-7-O-beta-D-apiosyl-4'-	Glycyrini-7-o-d-paracino-4	Flavonoids

						O-beta-D-glucoside	'-O-D-glucoside	
33	417.1188	4.82	0.2	[M-H]-	0.9607	Liquiritin	Glycyrrhizin	Flavonoids
34	623.1976	5.06	0.4	[M-H]-	0.9948	Verbascoside	Verbascoside	Phenylethanoids (C6-C2)+Phenylpropanoids (C6-C3)
35	609.182	5.38	0.5	[M-H]-	0.9949	Hesperidin	Hesperidin	Flavonoids
36	665.2086	6.03	0.1	[M-H]-	0.9414	6-Acetylacteoside	Acetylergosteroside	Phenylethanoids (C6-C2)+Phenylpropanoids (C6-C3)
37	493.2288	7.21	0.4	[M+HCOO]-	0.9684	(3R)-3, 7-dimethylocta-1,6-dien-3-yl 6-O-.alpha. -l-arabinopyranosyl.beta.-D-glucopyranoside	NA	Monoterpenoids
9	539.1766	7.56	0.6	[M+HCOO]-	0.9837	Harpagoside	Harpagoside	Monoterpenoids
38	241.0866	9.12	1.6	[M-H]-	0.9452	Lapachol	Yellow bellflower quinone	NA
39	281.1391	9.72	0.9	[M-H]-	0.9914	Octyl gallate	Octyl gallate	Phenolic acids (C6-C1)
40	207.102	9.86	3.0	[M-H]-	0.9033	3-(4-Isopropoxyphenyl)propanoic acid	4-isopropoxyphenylpropionic acid	NA
41	329.233	9.93	0.8	[M-H]-	0.9943	9-Octadecenoic acid, 5,8,11-trihydroxy-	5,8, 11-trihydroxy-9-octadecenoic acid	Octadecanoids
42	329.233	10.69	1.4	[M-H]-	0.9861	FA 18:1+3o	NA	Octadecanoids
43	821.3957	10.79	0.9	[M-H]-	0.9279	(2 s, 3 s, 4 s, 5 r, 6 r) - 6 - [(2 r, 3 r, 4 s, 5 s, 6 s) - 2 - [[(3 s, 6 ar, bs, as, 12 ar, 14 bs) - 11 - carboxy - 4 minus 2 a, 6 b, 8 a, 11, 14 b - heptamethyl - 14 - oxo - 2, 3, 4 a, 5,6,7,8,9,10,12,12 a, 14 a - h - picen dodecahydro - 1-3 - yl] foxy] - 6 - carboxy - 4, 5 -	NA	Triterpenoids

						dihydroxyoxan - 3 - yl] foxy - three, four, five - trihydroxyoxane - 2 - carboxylic acid		
44	367.1183	11.46	1.1	[M-H]-	0.9648	Glycycoumarin	Licorice coumarin	Coumarins
45	265.1231	12.27	1.5	[M-H]-	0.9997	Honokiol	And magnolol	Lignans
46	313.2381	12.42	0.7	[M-H]-	0.9624	12, 13 - DiHOME	NA	Octadecanoids
47	265.1231	12.87	0.0	[M-H]-	0.9999	Magnolol	Magnolol	Lignans
48	295.2275	13.5	1.5	[M-H]-	0.9908	12(13)-EpOME	NA	Fatty Acids and Conjugates
49	265.1476	13.74	0.9	[M-H]-	0.9327	Laurylsulfuric acid	NA	Fatty acyls
50	540.3303	13.97	0.8	[M+HCOO]-	0.976	LPC 16:0	NA	Glycerophospholipids

Note: (Header description: **NO**: serial number; m/z: mass-to-charge ratio of the parent ion; RT/min: Retention time /min; ppm: Grade 1 mass deviation; Adduct: Adduct; Score: Secondary mass spectrometry matching score; Compound EN: The English name of a compound; Compound CN: Chinese name of a compound; SuperClass: Compound classification; NA: Unavailable; * Identify compounds for reference substances.**)**

Add: 上海市闵行区园美路 58 号 1 号楼 1 building, 58 Yuan-mei Rd. Shanghai China 200233 | Tel: +86 021-5497 5031| E-mail: info_apt@sibs.ac.cn

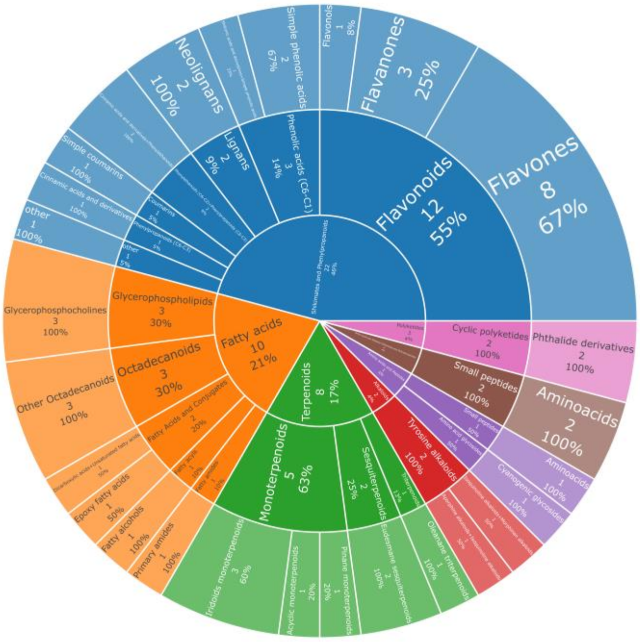

Figure 5 Proportion of each chemical classification of the peak chemicals in traditional Chinese medicine

Note: The classification of peak identified compounds in traditional Chinese medicine shows pathway, superclass top5, and class top3 categories

7. "Literature

[1] Hyun Woo Kim, Mingxun Wang, Christopher A Leber, et al, NPClassifier: A Deep Neural Network-Based Structural Classification Tool for Natural Products. J Nat Prod. 2021, 84 (11)

[2] Dunn, W.B., Erban, A., Weber, R.J.M., Creek, D.J., Brown, M., Breitling, R., Hankemeier, T., Goodacre, R., Neumann, S., Kopka, J., et al. Mass appeal: metabolite identification in mass spectrometry-focused untargeted metabolomics. Metabolomics,2012,9, 44-66.

[3] L, L., MH, R., B, P., X, S., S, C., H, R., JK, S., R, T., L, S., NC, L., et al. Metabolic Dynamics and Prediction of Gestational Age and Time to Delivery in Pregnant Women. Cell,2020,181(7) 1680-1692.

[4] Inamullah Hakeem Said , Johnathan Douglas Truex , Christina Heidorn , Mihella B Retta , Dimitar D Petrov , Sara Haka , Nikolai Kuhnert , et al. LC-MS/MS based molecular networking approach for the identification of cocoa phenolic metabolites in human urine. Food Research

International,2020,132.

[5] Sili Fan 1, Muhammad Shahid 2, Peng Jin 2, Arash Asher 3, Jayoung Kim , et al. Identification of Metabolic Alterations in Breast Cancer Using Mass Spectrometry-Based Metabolomic Analysis. Metabolites. 2020, 10(4): 170.

8. Attachment

1_ Attached figure: Figure 1_ BPC diagram of Changzhou Tongbian Fangzheng ion mode - Marked peak.pdf

2_ Illustration: Figure 2_ BPC in negative ion mode of Changzhou Tongbian - peak.pdf

3_ Attachment: Table 1_ Compound categories and quantite.xlsx

4_ Attached Figure: Figure 3_ proportion of all identified compounds in each chemical tax.pdf (.html)

5_ Figure: Figure 4_ Number proportion of peak Compounds of Traditional Chinese medicine in each chemical tax.pdf (.html)

6_ Attachment: Table 2_ Results of Chemical component.xlsx

9. Appendix: Methods in Chinese and English (for reference)

- **LC-MS Method**

CZTBF extracts were analyzed using a Vanquish UHPLC system (Thermo Scientific, Waltham, MA) equipped with a HSS-T3 column (100 x 2.1 mm, 1.8 µm particles size; Waters) at a column compartment nature of 35 ° C. Mobile phase A is H2O+0.1%formic acid and mobile phase B is acetonitrile+0.1%formic acid (LC-MS grade solvents, Fisher chemical. Samples were separated with a flow rate of 0.3 mL/min using the following gradient: 1 min isocratic at 5% B, up to 98 B in 16 min, back to 5% B in 0.5 min and then 2.5 min isocratic at 5% B.

Q-Exactive HFX mass spectrometer (Thermo Fisher Scientific, Bremen, Germany) was coupled

to the UHPLC system. Mass spectra were acquired in both electrospray ionization (ESI) positive and negative modes using data-dependent acquisition (DDA) modes with a mass range of m/z 90-1300. The MS/MS spectra were obtained from the top10 most intense MS1 ions. The stepped normalized high-energy dissociation (HCD) collision energies (CEs) of 20,40, and 60 units were used. Capillary temperature is 320 ° and probe heater temperature is 350 °.

■ **Database**

Compound annotation was done by matching accurate mass Youdaoplaceholder0 and MS/MS spectral to reference data from an in-house standards TCM database (Shanghai Applied Protein Technology CO., Ltd., Shanghai, China) and public databases GNPS (Wang, M.X.; Carver, J. J.; Phelan, V. V.; et al. Nat. Biotechnol. 2016, 34, 828−837. ReSpect (Sawada, Y. et al. Phytochemistry. 82, 38-45 (2012)) and Massbank (Horai, H.; Arita, M.; Kanaya, S.; et al. J. Mass Spectrom. 2010, 45, 703−714.
